# Understanding HIV-*Mycobacteria* synergism through comparative proteomics of intra-phagosomal mycobacteria during mono- and HIV co-infection

**DOI:** 10.1038/srep22060

**Published:** 2016-02-26

**Authors:** Rakesh Ganji, Snigdha Dhali, Arshad Rizvi, Srikanth Rapole, Sharmistha Banerjee

**Affiliations:** 1 Department of Biochemistry, School of Life Sciences, University of Hyderabad, Hyderabad, Telangana State, India; 2 National Centre for Cell Science, Pune, Maharashtra, India

## Abstract

*Mycobacterium tuberculosis* (*Mtb*) is the most common co-infection in HIV patients and a serious co-epidemic. Apart from increasing the risk of reactivation of latent tuberculosis (TB), HIV infection also permits opportunistic infection of environmental non-pathogenic mycobacteria. To gain insights into mycobacterial survival inside host macrophages and identify mycobacterial proteins or processes that influence HIV propagation during co-infection, we employed proteomics approach to identify differentially expressed intracellular mycobacterial proteins during mono- and HIV co-infection of human THP-1 derived macrophage cell lines. Of the 92 proteins identified, 30 proteins were upregulated during mycobacterial mono-infection and 40 proteins during HIV-mycobacteria co-infection. We observed down-regulation of toxin-antitoxin (TA) modules, up-regulation of cation transporters, Type VII (Esx) secretion systems, proteins involved in cell wall lipid or protein metabolism, glyoxalate pathway and branched chain amino-acid synthesis during co-infection. The bearings of these mycobacterial factors or processes on HIV propagation during co-infection, as inferred from the proteomics data, were validated using deletion mutants of mycobacteria. The analyses revealed mycobacterial factors that possibly via modulating the host environment, increased viral titers during co-infection. The study provides new leads for investigations towards *hitherto* unknown molecular mechanisms explaining HIV-mycobacteria synergism, helping address diagnostics and treatment challenges for effective co-epidemic management.

Resurgence of tuberculosis (TB), a curable infectious disease, with the pandemic of Human Immunodeficiency Virus (HIV), the virus that causes Acquired Immunodeficiency Syndrome (AIDS) is a major health issue worldwide^1^. The current statistics by WHO divulges that a third of HIV infected people worldwide are simultaneously infected with *Mycobacterium* species, mainly *Mycobacterium tuberculosis* (*Mtb*), the TB causing bacteria (WHO report, 2014). Disease management of mycobacterial infections in HIV patients is highly challenging because of drug to drug interactions, drug toxicity, increased incidence of TB related Immune reconstitution inflammatory syndrome (IRIS)^2^,^3^,^4^ and emerging threat from opportunistic infections by environmental mycobacteria. These mycobacteria are ubiquitously present in the environment including soil, water, air and food^5^. Some of the common opportunistic mycobacteria include *Mycobacterium avium* complex (MAC), *M. kansasii*, *M. fortuitum*, *M. gordonae*, *M. phlei*, *M. xenopi* and attenuated strains like *M. bovis* BCG (BCG)^5^,^6^. HIV patients with mycobacterial co-infections have been reported to progress to AIDS faster than those without co-infection^7^, at the same time, mycobacteria that can otherwise be cleared establish infection in HIV patients, pointing to a strong mutualism between these two pathogens^8^.

Mycobacteria reside inside the phagosomes of infected host macrophages. They adapt to the hostile intracellular conditions by concerted modulation of their protein expression and host signaling^9^. The adaptation of pathogenic mycobacterial proteome to the hostile intra-phagosomal environment has been studied using both transcriptomics and proteomics approaches^10^,^11^,^12^,^13^,^14^. These studies have been successful to a large extent in understanding the pathogenesis of TB bacteria. However, being restricted to mono-infection, these studies do not explain how a mycobacterium adapts to macrophages, where the cells have had a prior exposure to HIV. A population of monocytes/macrophages is colonized and used as reservoir by HIV during infection, but a large fraction of monocytes are also stimulated owing to immune environment generated by infected macrophages that makes them more aggressive phagocytes than usual^15^,^16^,^17^,^18^. Mycobacteria, during co-infection, encounters altered, quite possibly, activated macrophages as the host is already infected with HIV-1, whereas during mono-infection, mycobacteria faces the challenges within a naïve monocyte^19^. This prompts one to look at the changes in the early adaptive responses of mycobacteria to survive inside the phagosomes of infected macrophages during HIV-mycobacteria co-infections. Further, mycobacterial infection exacerbates HIV disease progression^20^ and it is not clear if mycobacterial factors or processes are directly or indirectly involved in increasing HIV titers during co-infection. In order to understand these two aspects of HIV-mycobacteria co-infection biology, we undertook a proteomics approach to understand the early adaptive changes in intraphagosomal mycobacterial protein in the phagosome-enriched fractions of HIV-mycobacteria co-infected macrophages.

The proteomics studies were performed using human derived macrophages THP-1 infected either with Bacille-Calmette-Guérin (BCG), a live attenuated strain of *Mycobacterium bovis* alone or in the background of M-tropic strain ADA-8 of HIV-1. This cell culture based set up simulated a condition when an HIV patient acquired secondary mycobacterial infection. In compliance with the clinical observations on HIV-mycobacterial co-infections, the co-infection of THP-1 with HIV and mycobacteria resulted in higher propagation of both the virus and the bacteria^21^.

In this paper, we discuss the differentially expressed intra-phagosomal mycobacterial proteins in response to mycobacterial mono-infection and HIV co-infection and correlate the same with mycobacterial survival and viral titers. The intra-phagosomal mycobacterial proteome suggested key alterations in the toxin-antitoxin modules, lipid metabolism, cation transporters and Type VII Esx secretory systems during co-infection. Some of the leads from the proteomics analyses were validated through deletion mutants or over-expressing mycobacterial strains that revealed involvement of factors from mycobacteria that possibly via modulating the host environment, promote HIV propagation during co-infection. The study is the first attempt to catalogue the early changes in the mycobacterial proteomes in the phagosomes of host cell during co-infection to understand the synergism between HIV and mycobacteria. Identification of mycobacterial proteins differentially expressed during mono- and co-infection will help us decipher the mycobacterial strategies employed to survive inside phagosomes of macrophages during co-infection and identify factors that have impact on viral propagation during co-infection.

## Results and Discussion

### Comparative proteomes of intra-phagosomal mycobacteria from mono- and HIV co-infected THP-1 macrophages

**Figure 1:**
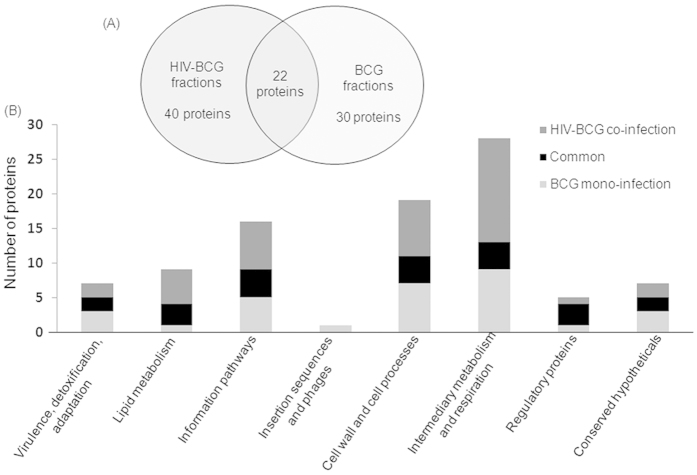
Functional categorization of mycobacterial proteins identified from the phagosome-enriched fractions of BCG mono- and HIV-BCG co-infected cells. (**A**) Venn diagram of mycobacterial proteins distributed between mono- and co-infections. (**B**) Graphical representation of functional categories of the proteins from mono- and co-infections.

**Figure 2:**
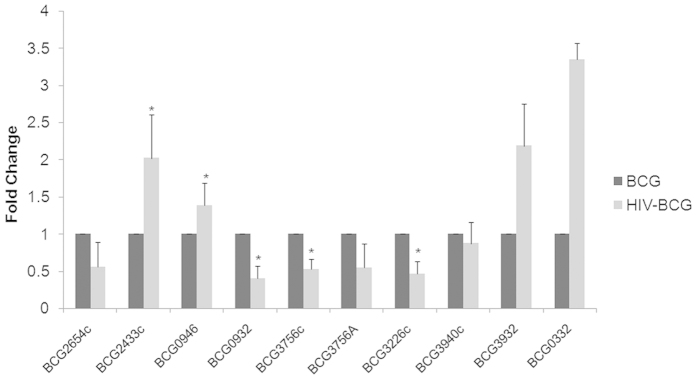
Real-time PCR of RNA from intracellular BCG reiterates the proteomic data. Statistical analyses were done with Student’s *t*-test. *represents p < 0.05.

We studied the early adaptive changes in the proteome of the intraphagosomal mycobacteria in the background of HIV infection. We used THP-1 derived macrophages which were either infected with *M. bovis* BCG (Multiplicity of infection (MOI) = 100) alone or co-infected with ADA-8 HIV-1 (at 30 ng/mL p24 equivalents) and BCG (refer methods). High MOI of 100 was used to enrich mycobacteria-laden phagosomes to identify the intra-phagosomally expressed mycobacterial proteins. To capture the early events, after 24 hr post-infection, we isolated phagosome-enriched fractions, to minimize the host background, from BCG mono- and HIV-BCG co-infected cells and performed LC-MALDI-MS/MS of the fractions. The results of proteomic data were from the three experiments of BCG mono-infected fractions and four experiments of HIV-BCG co-infected fractions. Proteins identified (95% confidence) from these experiments of a condition (mono- and co-infection) were pooled together into their respective categories. Then, the proteins identified in at the least two experiments per category were considered for further analyses. Overall, we have identified 92 differentially expressed proteins between BCG mono- and HIV-BCG co-infected fractions (Table 1, S1 and S2). Of these proteins, 22 proteins overlapped between both the fractions, 30 and 40 proteins were exclusively present in BCG mono-infected and HIV-BCG co-infected fractions, respectively (Table 1, S1, S2 and Fig. 1A). Proteins exclusively present in one condition but absent in the other condition were considered for further analyses. The proteins were categorized into different functional categories as described in *Tuberculist*^22^ database (Fig. 1B). The proteins belonging to two major groups, that is, lipid metabolism and intermediary metabolism and respiration, were upregulated during co-infection. The other group that was categorically enriched during co-infection was information pathways (Fig. 1B).The proteomic data were further validated by the Real-Time PCR of randomly picked 10 mycobacterial genes using the RNA isolated from the intraphagosomal BCG during BCG mono- and HIV-BCG co-infection (Fig. 2). The Real-Time PCR results reiterated the proteomic data, where BCG0946 and BCG3940c were common for both mono- and co-infections, BCG2654c, BCG0932, BCG3756c and BCG3226c were observed upregulated in mono-infection and BCG3932, BCG0332 and BCG2433c were observed upregulated in co-infection. Some of the important observations are discussed below. These observations are supported by co-infection studies with the knock-out/over-expression mutants of *M.smegmatis* to understand the synergistic impact of factors from mycobacteria on HIV propagation during co-infection.

### Reduced expression of Toxin-Antitoxin systems in mycobacteria during co-infection

**Figure 3:**
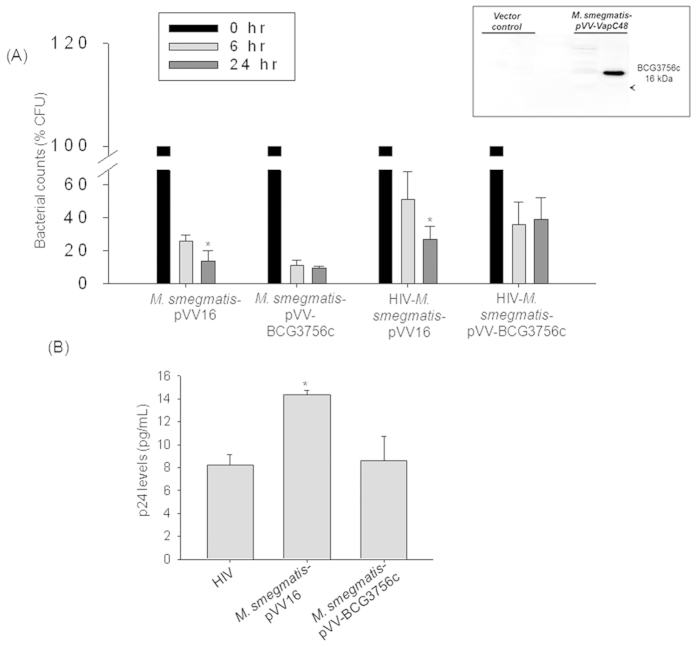
BCG3756c (VapC48) expression in *M.smegmatis* helps its survival without affecting HIV titers. (**A**) Plot representing *M.smegmatis-*pVV16 and *M.smegmatis-*pVV-BCG3756c survival inside macrophages upon mono- and co-infections in percentage CFU (%CFU) at 0, 6 and 24 hrs post-infection. *Inset:* Western blot with anti-His antibody confirming the expression His-tagged BCG3756c in *M.smegmatis*. (**B**) Bar graph represents HIV p24 titers in the supernatants of HIV mono-, HIV-*M.smegmatis-*pVV16 and HIV-*M.smegmatis-*pVV-BCG3756c co-infected macrophages at 24 hr post-infection. The HIV titers were not detectable in the culture supernatants at 6 hr post-infection.

During mono-infection, expression of proteins belonging to Virulence-detoxification-adaptation category such as BCG3756c (VapC48) and BCG3477 (VapB47) were upregulated. BCG3756c (toxin) and BCG3477 (anti-toxin) belong to Toxin-Antitoxin (TA) family of proteins^22^. Toxin-Antitoxin systems are expressed during stress and help in the viability of the bacteria by allowing the expression of stress responsive genes^23^. The up regulation of BCG3756c during mono-infection was validated by qRT-PCR (Fold change in transcript levels - BCG mono-infection: 1 ± 0.001 fold; HIV-BCG co-infection: 0.53 ± 0.135 fold; Fig. 2). As BCG3756c was over-expressed during BCG mono-infection, we expected that it may play a role in intracellular survival of mycobacteria. We expressed BCG3756c gene with C-terminus 6× His-tag in *M.smegmatis*, that does not have an ortholog of BCG3756c, using a mycobacterial shuttle vector, pVV16 (Fig. 3A inset). The recombinant *M.smegmatis*-pVV-BCG3756c was used for mono- and HIV co-infections and scored for CFU by plating on 7H10 agar plates after 0, 6 and 24 hours post-infection. Accordingly, the recombinant strain survived better over time, 6 hr to 24 hr, inside macrophages as compared to *M.smegmatis*-pVV16 both during mono- and co-infection. The clearance of recombinant strain *M.smegmatis*-pVV-BCG3756c (6 hr: 10.92 ± 3.21%CFU; 24 hr: 9.42 ± 1.04%CFU) was not significant from 6 hr to 24 hr (Fig. 3A) compared to the vector control, *M.smegmatis-*pVV16 (6 hr: 25.98 ± 3.39%CFU; 24 hr: 13.75 ± 6.25%CFU; p < 0.05) which was cleared by 45% from 6 hr to 24 hr time point during mono-infections (Fig. 3A). During co-infection, *M.smegmatis*-pVV-BCG3756c (6 hr: 35.64 ± 14.19%CFU; 24 hr: 38.95 ± 13.47%CFU) was not cleared efficiently from 6 hr to 24 hr (Fig. 3A) whereas *M.smegmatis-*pVV16 (6 hr: 51.31 ± 16.74%CFU; 24 hr: 26.78 ± 8.07%CFU; p < 0.05) was cleared by 48% from 6 hr to 24 hr time point. The percentage CFU was normalized to that of 0 hr. Evident from these results, over-expression of BCG3756c, irrespective of mono- or co-infections, helped in the survival of mycobacteria.

Since, BCG3756c was down-regulated during co-infection as per the proteomics data we scored the impact of over-expression of this protein on HIV titers during co-infection. It was observed that the co-infection with the Vector control increased the p24 equivalents of viral titers (HIV-*M.smegmatis-*pVV16: 14.36 ± 0.36 pg/mL; p < 0.05) compared to HIV infection (HIV: 8.21 ± 0.91 pg/mL) alone (Fig. 3B), but co-infection with the recombinant strain failed to provide such support to viral propagation (HIV-*M.smegmatis-*pVV-BCG3756c: 8.63 ± 2.12 pg/mL) as compared to HIV infection (HIV: 8.21 ± 0.91 pg/mL) (Fig. 3B). VapBC TA systems are considered as stress responsive elements which help the bacteria persist during stress probably by decreasing the metabolic activity and activating the required pathways. This function is probably carried out by its annotated ribonuclease activity on mRNAs and thus inhibiting the translation of avoidable proteins during stress^23^. During this process probably, toxin also inhibits the factors which can influence the viral titers. Therefore, expression of proteins belonging to TA systems helps in the persistence of mycobacteria upon infection but probably has no impact on HIV propagation during co-infection.

This explained the down-regulation of this factor in the proteomics data during co-infection.

### Mycobacterial iron dependent transcription regulator (IdeR) promoted viral titers during co-infection

Amongst the identified mycobacterial proteins upregulated during co-infection, BCG0332 (52% similar to MSMEG_0626), BCG1633 (76.5% similar to MSMEG_3200), BCG2957 (52% similar to MSMEG_0408), BCG2636c (78.5% similar to MSMEG_3200), BCG2962c (75% similar to MSMEG_4727) belonged to IdeR regulon (Table 1)^24^. IdeR (91% similar to MSMEG_2750) is a global iron dependent transcriptional factor that regulates the expression of several critical proteins in mycobacteria^24^. Hence, using the mutant, *M.smegmatis* (*M.smeg*Δ*ideR*) and complemented strain (*M.smeg*Δ*ideR*-pVV-IdeR)^25^, we checked the effect on mycobacterial survival and HIV titers during co-infection. IdeR complemented strain was prepared by expressing the IdeR gene cloned into pVV16 shuttle vector in the mutant, *M.smeg*Δ*ideR* (Figure S1).

**Figure 4:**
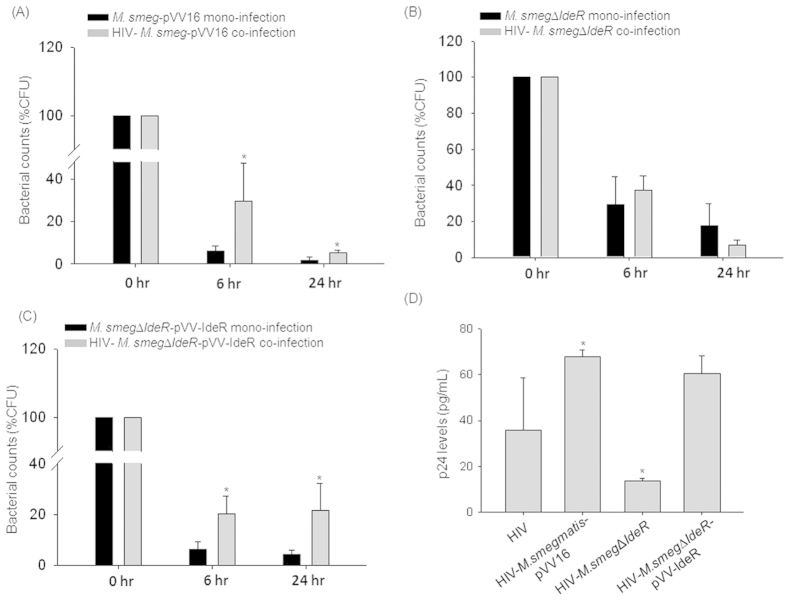
IdeR or IdeR dependent proteins support both mycobacteria and HIV titers during co-infection. (**A**) Plot representing (**A**) *M.smeg-*pVV16, (**B**) *M.smeg*∆*IdeR* and (**C**) *M.smeg∆IdeR-*pVV-IdeR survival inside macrophages upon mono- and co-infections in percentage CFU (%CFU) at 0, 6 and 24 hr post-infection. (**D**) Bar graph represents HIV p24 titers in the supernatants of HIV mono-, HIV-*M.smeg-*pVV16, HIV-*M. smeg∆IdeR* and HIV-*M. smeg∆IdeR*-pVV-IdeR co-infected macrophages at 24 hr post-infection. The HIV titers were not detectable in the culture supernatants at 6 hr post-infection.

HIV co-infection helped in the survival of the wildtype mycobacteria (*M.smegmatis-*pVV16) as observed in terms of percentage CFU (*M.smegmatis-*pVV16 mono-infection: 1.72 ± 1.59%CFU; co-infection: 5.14 ± 1.29%CFU at 24 hr) (Fig. 4A); however HIV co-infection could not support the survival of the IdeR mutant *M.smeg*Δ*ideR* (mono-infection: 17.88 ± 11.88%CFU; co-infection: 7.13 ± 2.36%CFU at 24 hr; Fig. 4B), which was rescued in the complement strain *M.smeg*Δ*ideR*-pVV-IdeR (mono-infection: 4.28 ± 1.87%CFU; co-infection: 21.56 ± 10.68%CFU at 24 hr; Fig. 4C), suggesting that IdeR is essential for HIV-supported survival of mycobacteria. The percentage CFU (%CFU) was normalized to 0 hr.

We then compared the p24 equivalent of viral titers during co-infection using all the three strains, *viz*; with the vector control, HIV-*M.smegmatis-*pVV16 (HIV: 35.97 ± 22.55 pg/mL; HIV-*M.smegmatis-*pVV16: 67.80 ± 2.94 pg/mL; p < 0.05), with the mutant, HIV-*M.smeg*Δ*ideR* (HIV-*M.smeg*Δ*ideR*: 13.76 ± 1.10 pg/mL; p < 0.05) and with the complemented strain, HIV-*M.smeg*Δ*ideR*-pVV-IdeR (HIV-*M.smeg*Δ*ideR*-pVV-IdeR: 60.37 ± 7.99 pg/mL) (Fig. 4D). We observed that the co-infection with the mutant strain decreased virus production as compared to the wild type. This decreased viral titers were restored upon complementation in the mutant (HIV: 35.97 ± 22.55 pg/mL; HIV-*M.smeg*Δ*ideR*-pVV-IdeR: 60.37 ± 7.99 pg/mL). The defects in the survival of the IdeR mutant and the drastic decrease in the HIV p24 titers during co-infection compared to HIV mono-infection or HIV-*M.smeg-*pVV16 co-infection suggested that IdeR is essential for mycobacterial survival and also involved in the increase of viral titers during co-infection. This could be a direct effect of IdeR or because of the proteins belonging to IdeR regulon. BCG2957 and BCG2636c are involved in the synthesis of cell wall lipids, phosphatidylinositol mannoside and lipoarabinomannans, respectively^26^,^27^,^28^. The alterations in the composition of mycobacterial cell wall lipids could alter the stimulation of the macrophages and thereby macrophage responses, which in turn can influence HIV propagation in a host^29^.

### Alterations in the mycobacterial cell wall and lipid metabolism influence viral production and mycobacterial survival during co-infection

*Mycobacterium* relies on fatty acids as a source of energy and probably synthesizes triacylglycerols as energy reserves during infections for long-term sustenance inside the host^30^,^31^. BCG proteome indicated expression of proteins that are involved in synthesis of Phthiocerol Dimycocerosates (PDIMs) during both mono- and co-infection conditions. PDIMs are key components of cell wall determining the virulence of mycobacteria^32^. These proteins included Long-chain-fatty-acid-CoA ligase FadD15/BCG2202, Mycocerosic acid synthase (BCG2962c), Phenolpthiocerol synthesis polyketide synthase PpsA/BCG2953 and probable enoyl-CoA hydratase echA6/BCG0957. However, Phenolpthiocerol synthesis polyketide synthase type I PpsE/BCG2957 (involved in the synthesis and translocation of PDIM to cell wall surface), Phosphatidylinositol mannoside acyltransferase/BCG2636c (involved in the synthesis of lipomannans and lipoarabinomannans) and Putative diacyglycerol O-acyltransferase/BCG3443 (involved in the synthesis of triacylglycerols)^26^,^27^,^28^ were upregulated during co-infection. A putative zinc metalloprotease (Rip1/BCG2891c), a protein known to regulate the cell envelope lipid composition important for the virulence of mycobacterium^33^ was over-expressed during HIV-BCG co-infection. Rip1 was also shown to positively regulate transcription of other proteins BCG2962c and BCG2953^34^, which concurrently appeared in our proteomics data (Table 1). Together the over-expression of BCG2891c, BCG2962c and BCG2953 can regulate the composition of cell wall affecting the virulence of the mycobacterium.

**Figure 5:**
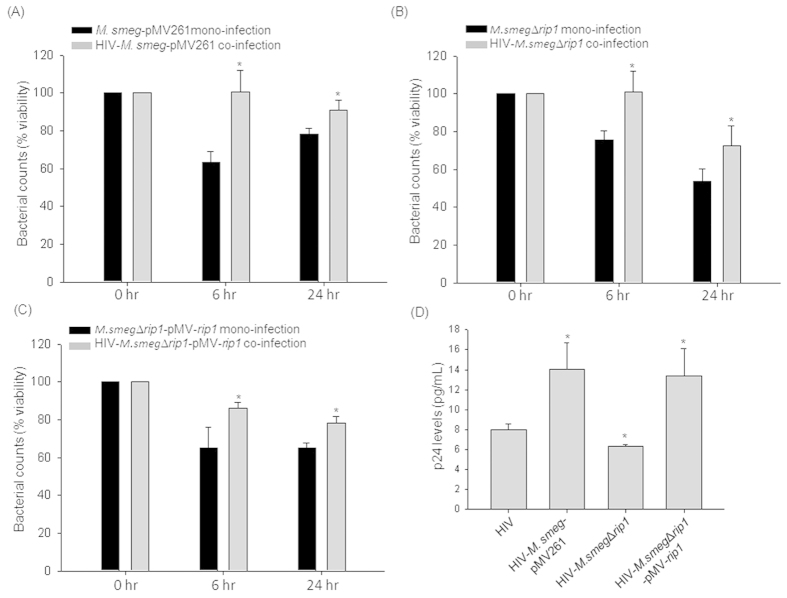
Mycobacterial Rip1 protein impacts the viral titers during co-infection. Plot representing (**A**) *M.smeg-*pMV261, (**B**) *M.smeg*∆*rip1* and (**C**) *M.smeg*∆*rip1-*pMV-*rip1* persistence inside macrophages upon mono- and co-infections in % viability (refer methods) at 0, 6 and 24 hr post-infection; (**D**) Bar graph represents HIV p24 titers in the supernatants of HIV mono-, HIV-*M.smeg-*pMV261, HIV-*M.smeg∆rip1* and HIV-*M.smeg∆rip1*-pMV-*rip1* co-infected macrophages at 24 hr post-infection. The HIV titers were not detectable in the culture supernatants at 6 hr post-infection.

The significance of the over-expression of mycobacterial proteins involved in the cell wall and lipid metabolism in the context of co-infection was studied next using *knock-out* (KO) mutants of *M. smegmatis* for the proteins, Rip1/MSMEG_2579 (81% similar to BCG2891c) and Polyketide synthase (Pks) (52% similar to BCG2957). As these proteins were over-expressed during co-infection, it was speculated that they may influence both mycobacterial survival and HIV titers during co-infection. The Pks was selected as it is reported to be involved in the synthesis of surface glycopeptidolipid (GPL) of mycobacteria^35^. THP-1 cells were either infected with mutants *M.smegmatis*::Δ*rip1* (Δ*rip1*)/ *M.smegmatis*::Δ*pks* (*M.smeg*Δ*pks*)^35^,^36^ alone or in the presence of HIV. As compared to mono-infection by vector control (*M.smeg*-pMV261), rip1 mutant (*M.smeg*Δ*rip1*) or the complemented strain (*M.smeg*Δ*rip1*-pMV*-rip1*), all the strains demonstrated increased survival upon co-infection with HIV (Fig. 5A–C). The comparative % viability upon mono- and co-infections at 24 hrs post mycobacterial infection are: *M.smeg*-pMV261: 78.10 ± 3.24% while HIV-*M.smeg*-pMV261: 91.14 ± 4.96% (Fig. 5A); *M.smeg*Δ*rip1*: 53.77 ± 6.69% while HIV-*M.smeg*Δ*rip1*: 72.48 ± 10.80% (Fig. 5B) and *M.smeg*Δ*rip1*-pMV*-rip1*: 64.98 ± 2.95% while HIV- *M.smeg*Δ*rip1*-pMV*-rip1*: 78.47 ± 3.20% (Fig. 5C). With this one can infer, that HIV background helped the survival of all the strains of mycobacteria, while deletion of *rip1* had no impact on survival. However, HIV-*M.smeg*Δ*rip1* co-infection showed significantly (p < 0.05) lesser p24 levels (6.35 ± 0.18 pg/mL) compared to HIV mono-infection (8.01 ± 0.57 pg/mL) and HIV-*M.smeg*-pMV261co-infection (14.07 ± 2.65 pg/mL) (Fig. 5D). The expression of Rip1 in *trans* in the mutant (*M.smeg*Δ*rip1-*pMV*-rip1*) restored the HIV titers equivalent to the co-infection with the vector control (13.38 ± 2.74 pg/mL) (Fig. 5D). These experiments indicated that directly or indirectly, mycobacterial factor Rip1 definitely augmented HIV production during co-infection.

**Figure 6:**
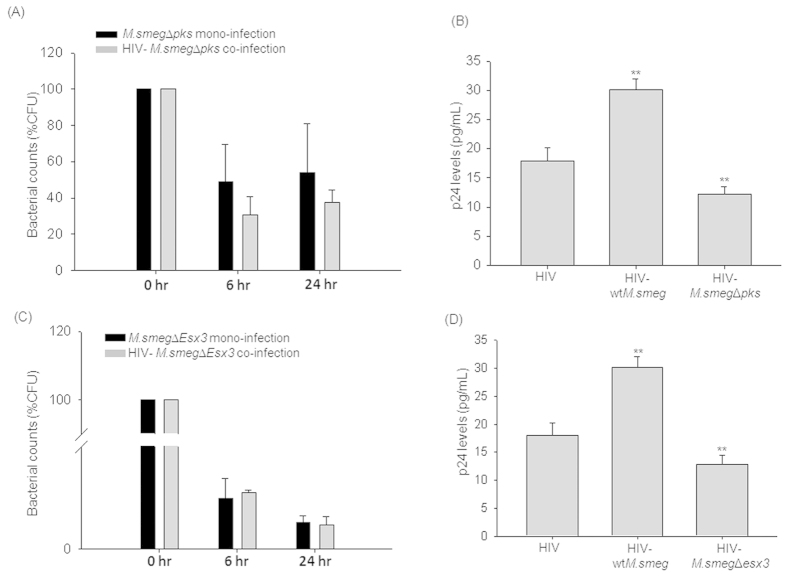
Validation of the inferences from the proteomics data using *M.smegmatis knock-out* (KO) mutants. The cells were infected with *M.smegmatis* KO mutants for mono-infections and along with HIV for co-infection to score for mycobacterial survival and viral titers. Percentage CFU (%CFU) of (**A**) *M.smeg*Δ*pks* and (**C**) *M.smeg*Δ*esx3* upon mono- or co-infection at 0, 6 and 24 hr post-infection, represented as bar plots. HIV p24 titers from the culture supernatants were measured at 24 hr post-infection and represented as bar graphs accordingly (**B**) *M.smeg*Δ*pks* and (**D**) *M.smeg*Δ*esx3*. The HIV titers were not detectable in the culture supernatants at 6 hr post-infection.

The Pks protein, involved in the synthesis of cell wall glycopeptidolipid, is a large multi-functional protein corresponding to 390 kDa with 11 active functional domains. The *M.smegmatis* pks mutant (*M.smeg*Δ*pks*) was used to score for the CFU and the impact on the viral titers. Percentage CFU of intracellular *M.smeg*Δ*pks* showed defect in survival during co-infection (37.68 ± 6.58%CFU at 24 hr) and mono-infection (54.21 ± 26.66%CFU at 24 hr) suggesting that it is required for the intracellular survival during co-infection (Fig. 6A). Similarly, HIV-Δ*pks* (12.24 ± 1.25 pg/mL) co-infection showed significantly (p < 0.001) lesser p24 levels compared to both HIV mono-infection (17.94 ± 2.22 pg/mL) and HIV-*wtM.smeg* co-infection (30.11 ± 1.87 pg/mL) (Fig. 6B). Due to the large size (390 kDa) of the Pks protein making a complement of the mutant was difficult. But, however, owing to the multi-functional domain structure of the Pks protein, it would be interesting to investigate the above impact on the viral titers by *M.smeg*Δ*pks* is due to the intact multi-functional Pks protein or due to individual domains, which is part of future investigations in the laboratory. One can thus infer that mycobacterial protein Pks supported both the survival of mycobacteria and viral titers during co-infection. Taken together, one may hypothesize that the differences in the cell envelope proteins or lipid composition of mycobacteria modulate host cell response, increasing viral propagation during co-infection. This corroborates the earlier findings that the HIV propagation is differentially regulated by mycobacteria in a strain-dependent manner which was attributed to the differences in the cell wall composition^29^.

### Increased expression of Esx system and cation transporter proteins in mycobacteria during co-infection and impact on viral titers

We observed increased expression of components of Esx system (EccE3/BCG0332 and EccC5/BCG1816) along with cation transporter proteins (MntH/BCG0976c and BCG3299) during co-infection as compared to mycobacterial mono-infection. Esx systems are Type VII secretion systems of mycobacteria and are implicated in both *in-vitro* and intracellular survival^37^. BCG0332 (EccE3) (52% similar to MSMEG_0626), a component of Esx-3 system, confirmed by qRT-PCR (Fig. 2) was upregulated by 3.35 ± 0.221 folds during co-infection. Esx-3 system has been reported to be essential for *in-vitro* growth^38^ and sensed iron and zinc availability^39^ in pathogenic mycobacteria. One may speculate that the Esx-3 secretion system may indirectly be involved in iron and zinc acquisition for survival inside macrophages where these ions are limiting. This speculation was further strengthened by the concomitant increased expression of cation transporters, MntH (orthologue of eukaryotic Nramp)^40^,^41^ and BCG3299 (probable cation transporter P type ATPase C)^42^ during co-infection. Similarly, EccC5/ BCG1816, an Esx-5 system protein was present during co-infection. Esx-5 system, in pathogenic mycobacteria, has been implicated in the secretion of PPE proteins, maintenance of cell wall integrity and modulation of macrophage response^43^,^44^ and helps the survival of intracellular mycobacteria.

To understand the significance of these observations in the context of HIV-mycobacteria co-infection, we used Esx-3 as representative system and studied the *M.smegmatis esx3* deletion mutant (*M.smeg*Δ*esx3*) where the whole cassette of *esx-3* system was disrupted^45^. We expected that Esx-3 system, whose component protein (EccE3/BCG0332) was over-expressed selectively during HIV-mycobacteria co-infections may play a role not only in the intracellular survival of the attenuated mycobacteria, but also in inducing HIV production. THP-1 cells were either infected with Δ*esx3* alone or co-infected with HIV. The cells were lysed after 0, 6 and 24 hrs of *M.smeg*Δ*esx3* infection and plated onto 7H10 agar plates and CFU were counted. The percentage CFU was observed to be nearly similar for both *M.smeg*Δ*esx3* mono-infection (3.86 ± 1.00%CFU at 24 hr) and HIV-*M.smeg*Δ*esx3* co-infection (3.50 ± 1.23%CFU at 24 hr) (Fig. 6C). With these observations, one can say that Esx-3 system may provide additional survival advantage to mycobacteria during co-infection. When HIV titers in the culture supernatants were compared between HIV-*wtM.smeg* co-infection and HIV-*M.smeg*Δ*esx3* co-infection, it was observed that deletion of *esx-3* reduced the mycobacterial induced increase in HIV titers by more than 50%. The viral titers in the culture supernatant for HIV mono-infection was 17.94 ± 2.22 pg/mL, which significantly increased in HIV-*wtM.smeg* co-infection to 30.11 ± 1.87 pg/mL, but HIV-*M.smeg*Δ*esx3* co-infection showed only 12.79 ± 1.69 pg/mL of viral titers (Fig. 6D). This provided an experimental evidence of factors (here, Esx-3 system) from mycobacteria involved in promotion of HIV propagation, explaining the alliance between HIV and mycobacteria.

A challenging but interesting quest would be to characterize the secretome of opportunistic mycobacteria either alone or in tandem with HIV co-infection, to identify the mycobacterial factors that make the macrophage environment more conducive, making these factors attractive for anti-mycobacterial drug targeting.

### Heightened Intermediary metabolism and respiration in BCG during co-infection to support intracellular survival

Proteins belonging to intermediary metabolism and respiration category such as Malate synthase G (BCG1872c), Isopropylmalate synthase (BCG3770), Acetolactate synthase (BCG3025c and BCG1855), L-Aspartate oxidase (BCG1633), Glycerol-3-phosphate dehydrogenase (BCG3331c), pyruvate kinase (BCG1655) and probable L-lysine-epsilon-aminotransferase (BCG3319c and BCG3354c) were upregulated in the phagosomal fraction of co-infected cells. Malate synthase along with isocitrate lyase are the unique enzymes of glyoxylate shunt pathway. Earlier reports suggested that the glyoxylate shunt pathway is upregulated in pathogenic mycobacteria during the persistent phase *in vivo* and is important for the virulence^46^,^47^,^48^. Glyoxylate pathway utilizes the C2 substrates from fatty acids, which are abundantly found in mammalian cells, without generating carbon dioxide and help in the persistence of mycobacteria during nutrient stress^46^. This was consistent with our observation of increased lipid body accumulation in host during co-infection^21^ and mycobacteria adaptation to the host niche by concomitant expression of malate synthase. Isopropylmalate synthase and Acetolactate synthase, which were upregulated during co-infection, are involved in the synthesis of branched chain amino acids, Leucine, Isoleucine and Valine which may provide survival advantage during co-infection^49^,^50^. Glycerol-3-phosphate dehydrogenase and pyruvate kinase help bacteria utilize glycerol as carbon source^51^. L-Aspartate oxidase (involved in the biosynthesis of NAD+)^52^ was expressed more during co-infection. NAD+ plays an essential role in cellular metabolism, given its involvement in almost all metabolic pathways; hence, biosynthesis of NAD+ can be used as a potential target against pathogenic bacteria^53^. Over-expression of these enzymes during co-infection may be supporting the survival of mycobacteria under co-infection conditions.

The above results clearly demonstrated that intra-phagosomal mycobacteria indeed adapted differently during mono- and co-infection and while adapting to the HIV induced-conducive intracellular niche of co-infected cells for persistence, the mycobacterial factors, possibly through influencing host responses, also influence the viral propagation.

## Conclusions

Proteomics approach to catalog changes in the pathogen proteome helped us understand possible molecular mechanisms that explain how mycobacterial factors may help HIV propagation during co-infection, mutually benefiting both the pathogens. The study, to our knowledge, is the first attempt to catalog the intracellular mycobacterial differential proteins during co-infection. Considering the challenges of proteome identification of intracellular mycobacteria, which allows preferential identification of abundant proteins, getting a high coverage has been observed to be problematic, as also evident from other studies^10^,^54^,^55^. Hence, though coverage of 92 proteins with 70 differentially expressed proteins was fairly informative, we substantiated the analyses of our data with mycobacterial KO and over-expression mutant strains. The key mechanisms like toxin-antitoxin systems, Type VII (Esx) secretory systems, cell wall and lipid metabolism pathways, intermediary metabolism and respiration were modulated during HIV co-infection. We also observed that mycobacteria tend to adapt to the intracellular niche provided by HIV during co-infection by altering its protein complement. For instance, concurrent to the increased host lipid accumulation inside macrophages during co-infection^21^, it increases the expression of lipid utilizing mycobacterial enzymes or pathways during co-infection. Another significant observation from the data is the increased expression of proteins involved in altering the lipid and protein composition of the cell wall. The differences in the cell wall alter the macrophage signaling pathways probably influencing the viral replication and propagation. This corroborates the observation that different clinical strains affect viral replication in a strain-dependent manner during co-infection which was attributed to the differences in the cell wall composition^29^. All the proteins identified in the current study have homologues in the virulent *Mtb* H37Rv strain (Table 1). Upon comparisons with the intracellular mycobacterial proteome from granuloma of guinea pigs infected with *Mtb* reported by Kruh *et al.* group^56^, our proteomic data has shown an overlap of only 22 proteins out of 92 (23.91%). Elucidating their role would help understand the pathogenesis of *Mtb* during co-infection. Thus, the leads from the study can be pursued to understand co-infection biology, helping decipher new intervention strategies and biomarker to overcome the synergism between HIV and mycobacteria.

## Materials and Methods

The cell lines used were HEK293T (Human embryonic kidney) cell line (NCCS, Pune) and THP-1 monocyte leukemia cell line (Cat#TIB-202, ATCC). Bacterial strains used were *Mycobacterium bovis* BCG, *M.smegmatis* mc^2^155, *M.smegmatis::*Δ*rip1* and *M.smegmatis::*Δ*rip1-*complemented (gift by Prof. Michael S. Glickman), *M.smegmatis*Δ*Esx3* (gift by Prof. Eric J. Rubin), *M.smegmatis*Δ*pks* (gift by Dr. Rajesh S. Gokhale) and *M.smegmatis*Δ*IdeR* (gift by Dr. Marcela Rodriguez)^25^,^35^,^36^,^45^. Macrophage tropic HIV (NL-ADA8 was gifted by Dr. Jayant Bhattacharya) was used. The THP-1 cell line was maintained in RPMI 1640 media (Invitrogen, USA) supplemented with 10% FBS (South American origin, Gibco, USA) and incubated at 37 °C with 5% CO_2_. The media was changed when the cells were 90% confluent. The 293T HEK cell line was maintained in DMEM media (Invitrogen, USA) supplemented with 10% FBS and incubated at 37 °C with 5% CO_2_. When the cells were 90% confluent, the cells were trypsinized with the Trypsin-EDTA (Sigma-Aldrich, USA) and washed with Phosphate buffered saline pH 7.4 (PBS) and fresh complete media was added.

The protocols of handling mycobacterial and HIV strains for co-infection in the laboratory of SB were approved by the Institutional Biosafety Committee No. UH/SLS/IBSC/Review/SB-R-11 and SB-R-14. All experiments were performed in the facilities (F-60 and F-70) approved for Mycobacterial and HIV cultures by University of Hyderabad Institutional Biosafety Committee under Department of Biotechnology, Govt. of India.

**Note:** The detailed protocol for Growth conditions of mycobacteria, Preparation and quantification of infectious HIV-1 (ADA8) particles, Infection of macrophages and phagosome isolation, Sample preparation and LC-MALDI-MS/MS Analyses, MALDI-TOF/TOF Analyses, Peptide Identification and Statistical Analyses have been described earlier^21^ and provided the same as supplementary text along with this manuscript. Kindly refer supplementary material.

### CFU enumeration and intracellular bacilli viability measurement

As previously described^21^, for the CFU assay, 24-well tissue culture plates carrying 0.2 million THP-1 macrophages per well was used. The mono- and co-infected cells after the incubation were washed thrice with PBS to remove any extracellular bacilli followed by lysis of macrophages with sterile water at 37 °C for 10 min. The lysates were diluted and were plated on 7H10 agar plates. The plates were incubated at 37 °C with 5% CO_2_ and 95% humidity for 2–3 weeks (for BCG) and 2–3 days (for *M.smegmatis*) and the colonies were enumerated and represented as percentage CFU which was normalized to CFU at 0 hr as 100%. The intracellular viability of *Mycobacterium* was measured using the Alamar blue assay^57^,^58^ in a 24-well plate. The fluorescence was read in a fluorescence plate reader with excitation at 530 nm and emission at 590 nm. % viability was calculated by normalizing the fluorescence intensity at 0 hr as 100%.

### Intracellular Mycobacterial RNA isolation

The monolayers of the infected cells after 24 h were washed thrice with PBS. The cells were lysed in sterile water for 10 min at 37 °C. The lysate was centrifuged at 1500 rpm for 5 min to remove cell debris. The bacterium from the supernatant was pelleted at high speed (10000 g) for 10 min and then the bacterial pellet was subjected to RNA isolation. The bacterial pellet was resuspended in Trizol and added to pre-chilled, acid washed 0.1 mm glass beads and lysed by bead beating - pulse on: 1 min and pulse off: 2 min on ice. Added glycogen to a final concentration of 200 μg/mL and kept at RT for 10 min. 1/10^th^ volume of chloroform was added and vortexed vigorously for 15 sec and kept at RT for 10 min. Centrifuged at 12000 rpm for 15 min at 4 °C and collected the upper aqueous layer. The RNA was precipitated using isopropanol in presence of glycogen (200 μg/mL) and pellet was washed with 75% ethanol. Air dried and resuspended in RNase free water. Prior to reverse transcription the RNA was subjected to DNase treatment to remove any residual DNA contamination. The DNase treated RNA was reverse transcribed using Superscript III Reverse Transcriptase (Invitrogen) with random hexamers as primer. The reverse transcribed RNA was used for Real-time PCR.

### Cloning and Expression of BCG3756c (VapC48) and IdeR gene into pVV16 shuttle vector

The BCG3756c gene was PCR amplified from BCG genomic DNA and *IdeR* gene was PCR amplified from *Mtb* H37Rv genomic DNA (Table S3). The mycobacterial shuttle vector, pVV16^59^,^60^, was amplified in *Escherichia coli* DH5α strain in Luria Bertani broth media with kanamycin at 50μg/mL. The vector, BCG3756c and IdeR inserts were double digested with NdeI and HindIII (Fermentas, Thermo Fisher Scientific Inc., USA). The digested insert and vector were ligated using T4 DNA ligase (Fermentas, Thermo Fisher Scientific Inc., USA) downstream of heat shock protein 60 (hsp60) promoter and transformed into *Escherichia coli* DH5α. The genes were cloned with C-terminus 6X Histidine tag. The recombinant plasmids carrying BCG3756c and IdeR were electroporated into *M.smegmatis* mc^2^155 and *M.smegmatis::*Δ*IdeR,* respectively. The transformants were picked and grown in 7H9 broth media at 37 °C and culture was harvested and lysed. Supernatant and cell pellet were separated and fractionated on SDS-PAGE followed by immunoblotting using anti-His antibody to confirm the expression of BCG3756c and IdeR. The growth curve for all the transformants was performed (Supplementary Figure 1B and 1C).

### Immunoblot

The Mycobacterial cell lysates and supernatants were fractionated on 10% SDS-PAGE, transferred to Nitrocellulose membrane using GE-Amersham Western wet-transfer apparatus. The membrane was blocked with 5% non-fat milk powder in PBS with 0.1% Tween-20 at RT for 2 hrs. The primary antibody, Anti-His tag mouse antibody (SantaCruz Biotechnology Inc., USA) was prepared in PBS-T (PBS + 0.1% Tween-20) with 2% BSA 1:1000 dilutions, was added and incubated at 4 °C on platform rocker overnight. After 3 PBS-T washes, added the Anti-mouse HRP-conjugated secondary antibody (Santa cruz Biotechnology Inc., USA), diluted 1:2000 in PBS-T with 2% BSA, incubated at RT for 2 hrs. After 3 washes with PBS, ECL substrate (Femtolucent Plus-HRP, chemiluminiscent reagent from G-biosciences, USA) was added to the membranes and was scanned for chemiluminiscence using Versadoc Imaging system (Biorad).

### Real-time PCR

Equal amount of RNA was considered for equal loading and the corresponding cDNA was used for RT-PCR. As endogenous controls, 16S rRNA gene was used (Table S3). The 2X SyBr green mix (Takara Bio Inc., Japan) was used. The real-time PCR data analyses were done using the 2^−ΔΔCt^ method with endogenous 16S rRNA gene as control. The fold change in the transcript levels were calculated with respect to the BCG mono-infection. All the experiments were performed more than three times. For genes, BCG3932, BCG0332 and BCG3940c data is representative of two experiments.

## Additional Information

**How to cite this article**: Ganji, R. *et al.* Understanding HIV-*Mycobacteria* synergism through comparative proteomics of intra-phagosomal mycobacteria during mono- and HIV co-infection. *Sci. Rep.* **6**, 22060; doi: 10.1038/srep22060 (2016).

## Supplementary Material

Supplementary Information

## Figures and Tables

**Table 1:** Mycobacterial proteins identified by LC-MALDI-MS/MS from the phagosomal fractions of BCG mono- and HIV-BCG co-infected cells.

S#	BCG gene#	pI	MW (kDa)	Functional category	Product/Function (Known/probable/annotated)	Ortholog in *M.tuberculosis* H37Rv (Rv*#*)
	**Proteins Observed in fractions from BCG mono-infected cells**					
1	BCG1194c	4.9	81.5	intermediary metabolism and respiration	Involved *de novo* biosynthesis of Methionine	Rv1133c
2	BCG3477	10.7	11	virulence, detoxification, adaptation	Unknown; Possible Anti-toxin VapB47	Rv3407
3	BCG3226c	6.1	116.6	information pathways	Probable ATP-dependent DNA helicase/Has both ATPase and helicase activities	Rv3201c
4	BCG0136c	7.3	77.4	cell wall and cell processes	Probable cation-transporter P-type ATPase B CtpB/Cation-transporting ATPase	Rv0103c (ctpB)
5	BCG0422c	4.9	92.5	virulence, detoxification, adaptation	Probable endopeptidase ATP binding protein (chain B) ClpB (ClpB protein) (heat shock protein F84.1)	Rv0384c
6	BCG2367c	6.1	46.8	intermediary metabolism and respiration	Probable dGTP triphosphohydrolase	Rv2344c
7	BCG0677c	6.3	118.7	information pathways	Probable exonuclease V (beta chain) RecB/Involved in homologous recombination.	Rv0630c
8	BCG2238c	7.5	109.1	intermediary metabolism and respiration	Glutamate-ammonia-ligase adenylyltransferaseGlnE/Regulatory protein involved in the regulation of glutamine synthetase activity	Rv2221c
9	BCG2457c	5.4	74.6	intermediary metabolism and respiration	Glutamine-dependent NAD(+) synthetaseNadE (NAD(+) synthase/Involved in biosynthesis of NAD	RV2438c
10	BCG1867	5.3	99.4	intermediary metabolism and respiration	Probable glycine dehydrogenase GcvB/The glycine cleavage system catalyses the degradation of glycine.	Rv1832
11	BCG1419c	5.9	33.9	conserved hypotheticals	Conserved hypothetical protein/Unknown	Rv1357c
12	BCG2119	6.5	58.9	insertion seqs and phages	Conserved hypothetical protein/Unknown	Rv2100
13	BCG0079c	7.5	30.8	cell wall and cell processes	Possible membrane protein/Unknown	Rv0048c
14	Mb2002c	4.5	24.8	cell wall and cell processes	Immunogenic protein Mpt64/Unknown.	Rv1980c
15	BCG3662c	10.69	12	information pathways	Iron-regulated H-NS-like protein Lsr2/Has DNA-bridging activity.	Rv3597c
16	BCG3174	5.2	85.3	intermediary metabolism and respiration	Probable NADH dehydrogenase I (chain G) NuoG/Involved in aerobic|anaerobic respiration	Rv3151
17	BCG1088	6.3	74.6	cell wall and cell processes	Probable potassium-transporting P-type ATPase B chain KdpB	Rv1030 (kdpB)
18	BCG1391c	6.5	70.1	information pathways	Probable ATP-dependent helicase DinG/Probable helicase involved in DNA repair and perhaps also replication.	Rv1329c
19	BCG1182	4.99	52.1	intermediary metabolism and respiration	Probable glucose-6-phosphate 1-dehydrogenase Zwf1 (G6PD)/Involved in pentose phosphate pathway (first step)	Rv1121
20	BCG3756c	9.6	16.3	virulence, detoxification, adaptation	Possible toxin VapC48/Unknown	Rv3697c
21	BCG0453c	6.1	23.2	intermediary metabolism and respiration	Thiamine-phosphate pyrophosphorylaseThiE/Involved in thiamine biosynthesis	Rv0414c
22	BCG3326; BCG3362	8.7	28.5	information pathways	Probable endonuclease VIII Nei/Involved in damage reversal of DNA	Rv3297
23	BCG1573	5	63.1	lipid metabolism	Probable fatty-acid-AMP ligase FadD25/Function unknown, but involvement in lipid degradation	Rv1521
24	BCG0932	10.9	15.5	Regulatory proteins	Possible transcriptional regulatory protein (possibly MarR-family)/Thought to be involved in transcriptional mechanism.	Rv0880
25	BCG1357	5.4	65.1	information pathways	Probable transcription termination factor Rho homolog/Facilitates transcription termination by a mechanism that involves rho binding to the nascent RNA, activation of rho’S RNA-dependent ATPase activity, and release of the mRNA from the DNA template	Rv1297
26	BCG0937	8.7	39.7	conserved hypotheticals	Conserved hypothetical protein/Unknown	Rv0885
27	BCG0788	5.2	19.1	conserved hypotheticals	Conserved protein/Unknown	Rv0738
28	BCG2654c	10	46.2	conserved hypotheticals	Conserved protein/Unknown	Rv2627c
29	BCG0489c*	7.11	105.2	cell wall and cell processes	Probable conserved transmembrane transport protein MmpL4/Unknown. Thought to be involved in fatty acid transport.	Rv0450c
30	BCG2361; BCG2362	6.5	89.9	cell wall and cell processes	Probable conserved transmembrane transport protein MmpL9/Unknown. Thought to be involved in fatty acid transport.	Rv2339
	**Proteins Observed in fractions from HIV-BCG co-infected cells**					
1	BCG3770	4.78	70	intermediary metabolism and respiration	2-isopropylmalate synthase LeuA/Involved in leucine biosynthesis (at the first step)	Rv3710
2	BCG3025c	6.5	66	intermediary metabolism and respiration	Acetolactate synthase (large subunit) IlvB1/Involved in valine and isoleucine biosynthesis (at the first step)	Rv3003c
3	BCG0626	10.5	7.5	virulence, detoxification, adaptation	Possible antitoxin VapB26/Unknown	Rv0581
4	BCG0391	8.2	41.3	virulence, detoxification, adaptation	Probable chaperone protein DnaJ1/Acts as a co-chaperone.	Rv0352
5	BCG2216c	7.97	40.4	intermediary metabolism and respiration	Probable transmembrane cytochrome C oxidase (subunit II) CtaC/Involved in aerobic respiration.	Rv2200c
6	BCG3007c	12.4	22.1	Information pathways	DNA-binding protein HU homolog HupB (histone-like protein)/This protein belongs to the histone like family of prokaryotic DNA-binding proteins which are capable of wrapping DNA to stabilize it, and prevent its denaturation under extreme environmental conditions.	Rv2986c
7	BCG0006; BCG0036	5.2	92.2	Information pathways	DNA gyrase (subunit A) GyrA	Rv0006c
8	BCG3932	7.7	51	cell wall and cell processes	ESX conserved component EccB1/Unknown.	Rv3869
9	BCG0332*	10.5	35.9	cell wall and cell processes	ESX conserved component EccE3. ESX-3 type VII secretion system protein/Unknown.	Rv0292
10	BCG3331c	6.7	62	intermediary metabolism and respiration	Probable glycerol-3-phosphate dehydrogenase GlpD2/Involved in aerobic respiration and oxidation of glycerol.	Rv3302c
11	BCG3470	5.6	87	intermediary metabolism and respiration	Conserved protein/Function unknown; probably enzyme involved in cellular metabolism.	Rv3401
12	BCG2921c	7.7	84.5	intermediary metabolism and respiration	Possible formate dehydrogenase H FdhF/Decomposes formic acid to hydrogen and carbon dioxide under anaerobic conditions in the absence of exogenous electron acceptors	Rv2900c
13	BCG0535c	7.3	35.5	conserved hypotheticals	Conserved protein/Function Unknown.	Rv0493c
14	BCG2433c	6.5	28.4	conserved hypotheticals	Conserved protein/Function Unknown.	Rv2417c
15	BCG1633*	6.7	53.7	intermediary metabolism and respiration	Probable L-aspartate oxidase NadB/Quinolinate biosynthesis.	Rv1595
16	BCG1872c	4.8	80.4	intermediary metabolism and respiration	Malate synthase G GlcB/Involved in glyoxylate bypass (second step), an alternative to the tricarboxylic acid cycle	Rv1837c
17	BCG1689	4.9	88	information pathways	Probable phenylalanyl-tRNAsynthetase, beta chain PheT/Charging PHE-tRNA	Rv1650
18	BCG0447	5.04	72.9	intermediary metabolism and respiration	Probable phosphate acetyltransferasePta/Involved at the last step (of two) in the conversion of acetate to acetyl-CoA	Rv0408
19	BCG2957*	5.5	158	lipid metabolism	Phenolpthiocerol synthesis type-I polyketide synthase PpsE/Involved in phenolpthiocerol and phthioceroldimycocerosate (dim) biosynthesis:	Rv2935
20	BCG1855	5.7	57.2	intermediary metabolism and respiration	Probable acetolactate synthase IlvG/Valine and isoleucine biosynthesis (first step).	Rv1820
21	BCG3661c	5.6	93.5	intermediary metabolism and respiration	Probable ATP-dependent protease ATP-binding subunit ClpC1/Hydrolyses proteins in presence of ATP.	Rv3596c
22	BCG3299	7.4	76.5	cell wall and cell processes	Probable metal cation-transporting P-type ATPase C CtpC/Metal cation-transporting ATPase	Rv3270
23	BCG0957	6.3	26	lipid metabolism	Possible enoyl-CoA hydratase EchA6/Could possibly oxidize fatty acids using specific components	Rv0905
24	BCG0976c	10.7	44.9	cell wall and cell processes	Divalent cation-transport integral membrane protein MntH (BRAMP)/H(+)-stimulated, highly selective, divalent cation uptake system.	Rv0924c
25	BCG2596	6.5	28.1	conserved hypotheticals	Conserved protein/Function Unknown.	Rv2573
26	BCG3443	9.7	48.8	lipid metabolism	Possible triacylglycerol synthase/May be involved in synthesis of triacylglycerol	Rv3371
27	BCG0992	7.8	83.5	Information pathways	ATP dependent DNA ligase LigD/Involved in DNA double-strand break repair, by nonhomologous end joining (NHEJ).	Rv0938
28	BCG1479	8.4	24.2	cell wall and cell processes	Probable lipoprotein LprH/Function Unknown	Rv1418
29	BCG1655	5.3	50.5	intermediary metabolism and respiration	Probable pyruvate kinase PykA/Produces phosphoenol pyruvate in glycolysis	Rv1617
30	BCG2858c	5.3	18.9	information pathways	Probable ribosome-binding factor aRbfA/Associates with free 30S ribosomal subunits (but not with 30S subunits that are part of 70S ribosomes or polysomes).	Rv2838c
31	BCG2870c	8.5	66.9	intermediary metabolism and respiration	Possible magnesium chelatase/Function unknown; possibly introduces a magnesium ion into specific substrate/compound.	Rv2850c
32	BCG1671	4.7	78	information pathways	Probable excinuclease ABC (subunit B - helicase) UvrB/Involved in nucleotide excision repair.	Rv1633
33	BCG1667	4.78	98.4	information pathways	Probable DNA polymerase I PolA/Involved in post-incision events.	Rv1629
34	BCG3633c	5.96	33.58	intermediary metabolism and respiration	3,4-DHSA dioxygenase/Catalyzes the extradiol cleavage of 3,4-dihydroxy-9,10-seconandrost-1,3,5(10)-triene-9,17-dione (3,4-DHSA)	Rv3568c
35	BCG1816	5.1	152.8	cell wall and cell processes	ESX conserved component EccC5. ESX-5 type VII secretion system protein/Function Unknown	Rv1783
36	BCG2636c*	8.96	35.16	lipid metabolism	Probable acyltransferase/Catalyzes the acylation of the 6-position of the mannose residue linked to position 2 of the myo-inositol in phosphatylinositol mono- and DI-mannosides.	Rv2611c
37	BCG3639	9.4	21.9	regulatory proteins	Transcriptional regulatory protein KstR (probably TetR-family)/Involved in transcriptional mechanism. Predicted to control regulon involved in lipid metabolism.	Rv3574
38	BCG2891c	8.69	42.9	cell wall and cell processes	Membrane bound metalloprotease/Controls membrane composition	Rv2869c
39	BCG0602	10.63	41.24	lipid metabolism	MannosyltransferaseMgtA/Involved in lipomannan (LM) biosynthesis	Rv0557
40	BCG0733	4.69	77.20	Information pathways	Probable elongation factor G FusA1 (EF-G)/This protein promotes the GTP-dependent translocation of the nascent protein chain from the A-site to the P-site of the ribosome.	Rv0684
	**Proteins Observed in fractions from both mono- and co-infected cells**					
1	BCG1389c	5.3	78.6	intermediary metabolism and respiration	Unknown; probably involved in polysaccharide degradation	Rv1327c
2	BCG1368	4.7	59.2	intermediary metabolism and respiration	Probable ATP synthase alpha chain AtpA	Rv1308
3	BCG1370	4.5	53.09	intermediary metabolism and respiration	Probable ATP synthase beta chain AtpD	Rv1310
4	BCG1627	4.4	37.5	intermediary metabolism and respiration	Probable biotin synthetase BioB/ Involved in biotin synthesis.	Rv1589
5	BCG0389	4.5	66.8	virulence, detoxification, adaptation	Probable chaperone protein DnaK (heat shock protein 70)/Acts as chaperone	Rv0350
6	BCG2562c	6.5	41.7	intermediary metabolism and respiration	Probable chorismate synthase AroF/Involved in the synthesis of chorismate	Rv2540c
7	BCG2944c	4.9	130.6	cell wall and cell processes	Probable chromosome partition protein Smc/Plays an important role in chromosome structure and partitioning	Rv2922c
8	BCG0717	6	146.7	information pathways	DNA-directed RNA polymerase (beta’ chain) RpoC	Rv0668
9	BCG0716	4.67	129.2	information pathways	DNA-directed RNA polymerase (beta chain) RpoB	Rv0667
10	BCG3940c	10.6	57.6	cell wall and cell processes	ESX conserved component EccE2. ESX-2 type VII secretion system protein/Function unknown	Rv3885
11	BCG0946	7.2	42.7	Regulatory proteins	Possible transcriptional regulatory protein (possibly LuxR-family)/Thought to be involved in transcriptional mechanism.	Rv0894
12	BCG2301c; BCG2302c	6.3	25.4	conserved hypotheticals	Possible membrane protein/Unknown	Rv2286c
13	BCG2962c*	4.8	224	lipid metabolism	Probable multifunctional mycocerosic acid synthase membrane-associated Mas/Catalyzes the elongation of N-fatty acyl-CoA with methylamalonyl-CoA (not malonyl-CoA) as the elongating agent to form mycocerosyl lipids	Rv2940c
14	BCG1539	9.3	49.8	virulence, detoxification, adaptation	Peptidoglycan hydrolase/Unknown	Rv1477
15	BCG2953	5	198.8	lipid metabolism	Phenolpthiocerol synthesis type-I polyketide synthase PpsA/Involved in phenolpthiocerol and phthioceroldimycocerosate (dim) biosynthesis	Rv2931
16	BCG2112c	7.4	99.57	information pathways	ATP-dependent DNA helicase HelY/DNA helicase activity	Rv2092c
17	Mb2007c	9.3	32.8	Regulatory proteins	Probable transcriptional regulatory protein (probably LysR-family)/Involved in transcriptional mechanism.	Rv1985c
18	BCG1085c	5.8	92.7	Regulatory proteins	Probable sensor protein KdpD/Member of the two-component regulatory system KDPD/KDPE involved in the regulation of the KDP operon.	Rv1028
19	BCG0123	10.6	27.8	cell wall and cell processes	Possible membrane protein/Unknown	Rv0090
20	BCG1550	6.1	41.2	cell wall and cell processes	Possible exported conserved protein/Unknown	Rv1488
21	BCG3215c	9.2	107.4	cell wall and cell processes	Probable conserved transmembrane protein/Unknown	Rv3193c
22	BCG2500c	8.7	88.2	lipid metabolism	Probable glycerol-3-phosphate acyltransferase PlsB2/Involved in phospholipid biosynthesis	Rv2482c

*Note:* *Corresponding protein belongs to IdeR regulon^24^.
