# Supplementary material for: Understanding HIV-Mycobacteria synergism through comparative proteomics of intra-phagosomal mycobacteria during mono- and HIV co-infection
Source: Sci Rep. 2016 Feb 26;6:22060. doi: 10.1038/srep22060 (PMC4768096; doi:10.1038/srep22060)
Supplement: Supplementary Information [file srep22060-s1.pdf]

Understanding HIV-*Mycobacteria* synergism through comparative proteomics of intra-phagosomal mycobacteria during mono- and HIV co-infection

Rakesh Ganji[a], Snigdha Dhali[b#], Arshad Rizvi[a#], Srikanth Rapole[b], Sharmistha Banerjee[a*]

[a]Department of Biochemistry, School of Life Sciences, University of Hyderabad, Hyderabad, Telangana State, India.
[b]National Centre for Cell Science, Pune, Maharashtra, India.

#Authors have contributed equally.

*To whom correspondence should be addressed:
Dr. Sharmistha Banerjee, Department of Biochemistry, School of Life Sciences, University of Hyderabad, Hyderabad, Telangana State, India-500046
Tel: (+91)-40-66794573; Fax: (+91)-40-23010120 Email: sbsl@uohyd.ernet.in

Methods:

Growth conditions of *Mycobacteria*:

The mycobacteria were plated on 7H10 agar media (Hi-media, India) supplemented with 10% Oleic acid, Albumin, Dextrose and Catalase (OADC, Hi-Media, India). The plate was incubated at 37°C for 15 days. The colonies were picked into the 7H9 broth media supplemented with 10% OADC and the broth culture was incubated at 37°C at 180 rpm until the $OD_{600\ nm}$ reached 0.8 to 1. The culture was checked for any contamination using Ziehl-Neelsen (ZN) staining procedure. Prior to the infection, centrifuged the bacterial suspension at 200 ×*g* for 10 min and washed the bacterial pellet with PBS and then with RPMI 1640 media. Suspension of single-bacillus was obtained by passing the suspension through 26G 1 mL syringe for 10-20 times and then withdrew from the top of the tube.

Preparation and quantification of infectious HIV-1 (ADA8) particles:

Infectious HIV-1 particles were prepared by transfection of proviral DNA pNL-ADA8 into HEK293T cells by calcium phosphate method. Culture supernatants were collected at intervals of 24 hours post-transfection, filtered through 0.45 µM syringe filter (Millipore, USA), precipitated using Polyethylene glycol (Kutner, Zhang et al. 2009; Banerjee, Benjamin et al. 2014) and quantified by HIV-1 p24 ELISA kit (Advanced BioScience Laboratories Inc, USA) according to the manufacturer's protocol. Cells were infected with 30 ng/mL of p24 equivalents of HIV.

Infection of macrophages and phagosome isolation:

THP-1 cells were PMA (10 ng/mL) differentiated to macrophages in RPMI 1640 media with 10% FBS in 150 mm culture dishes. After 24 hrs the macrophages were washed, fresh media was added and kept for 48 hrs rest. The macrophages were divided into two categories 1) for co-infection: First infected with HIV for 2 hrs in incomplete RPMI media and followed by 24 hr incubation in complete RPMI media. This was followed by infection with BCG at an MOI=100 for 4 hrs which was modified from the protocol described earlier (Lee, Jethwaney et al. 2010). The cells were extensively washed to remove the extracellular mycobacteria and also HIV particles to ensure that the differences observed in mycobacterial counts or HIV titers was due to mycobacterial mono- or co-infection. 2) For mono-infection: The cells were treated similar to co-infection but without virus and followed by BCG infection. After 4 hrs of infection, fresh media with antibiotic was added and incubated for 24 hrs. The phagosomes enter late endosome state at 24 hr post infection. After 24 hrs the cells were harvested for fractionation to obtain enriched bacteria-*laden* phagosomes which was standardized in our laboratory using sucrose density gradient method as described earlier (Wandy Beatty and Russell 2001).

Sample preparation and LC-MALDI-MS/MS Analyses:

Total 500 µg of phagosome protein was trypsinized in each category (BCG *laden* phagosomes and HIV-BCG *laden* phagosomes) in presence of 0.1% SDS overnight at 37°C. The sample was then subjected to detergent removal using detergent removal spin columns (Thermo scientific Pierce, USA). A total of 2.5 µg digested peptides were separated using a Tempo Nano MDLC system (Eksigent) with 0.5 mm × 2 mm CapTrap™ C18 PepMap guard column (MichromBioresources, Auburn CA) and an Agilent 300 SB C18 0.075 µm × 150 mm, 3.5 µm, 300 Å Nano column (Agilent Technologies). The trap column was washed for 40 min with 0.1%

aqueous TFA/2% Acetonitrile (ACN) at a flow rate of 15 μL/min. The Nano LC system was operated at a flow rate of 300 nL/min. Desalted peptides were eluted using 98% water/2% ACN/0.1% TFA (Buffer A) and 2% water/98% ACN/0.1% TFA (Buffer B) with the following gradient: 5−50% B in 115 min, increase to 90% B in 12 min, decrease to 5% B in 13 min and equilibration at 5% B for 10 min. The eluent was mixed with MALDI matrix at 800 nL/min flow rate solution (2.5 mg/mL α-cyano-4-hydroxycinnamic acid [CHCA] in 80% ACN + 0.1% TFA) using a Ekspot Spotter (Eksigent) and spotted onto 28 × 44 spot arrays on 123 mm × 81 mm Opti-TOF LC/MALDI inserts (AB SCIEX). Spotting time was from 15 to 150 minutes, with 20 s spot intervals.

MALDI-TOF/TOF Analyses:

MS spectra were acquired on an AB SCIEX MALDI-TOF/TOF 4800 mass spectrometer equipped with a 200 Hz repetition rate Nd: YAG laser in positive ion mode in the mass range of 800−4000 m/z and 900 laser shots accumulation. MS spectra were internally calibrated using 4700 Proteomics Analyzer Standards Kit containing 6 peptide mix to 50 ppm accuracy. Laser intensity was set to 4000 for MS and 4600 for MS/MS acquisition. Precursor selection for MS/MS analysis was done by the 4000 Series Explorer Software (AB SCIEX, Darmstadt, Germany) in the strongest precursor first selection order using the following criteria: minimum S/N ratio, 60; maximum precursors per spot, 18. All MS/MS spectra were acquired with 2 kV collision energy by accumulation of 2500 laser shots.

Peptide Identification:

The data was analysed using GPS Explorer software (AB SCIEX) using MASCOT search engine in *Mycobacterium tuberculosis* complex taxonomy with following parameters: MS tolerance: 150ppm; MS/MS tolerance: 0.5Da; Enzyme used: Trypsin; Fixed modification: carbamidomethyl; Variable modification: oxidation (methionine), Deamidation (N,Q). We have reported only those proteins with a protein identification confidence interval of ≥95% ($p<0.05$).

Statistical Analyses:

All the experiments were performed at the least three times. The data were analysed using SigmaPlot software version 11.0.0.77 (Systat Software, Inc., USA). The error bars represent the standard deviation (SD) from the mean of at least three independent experiments. Statistical analyses of the experimental data for comparing two groups were performed by Student's *t*-test. $p<0.05$ was considered as significant. * in the figures represent $p<0.05$ and ** represents $p<0.001$.

Supplementary data:

Figure S1:

Western blot using anti-His antibody confirming the expression His-tagged IdeR in *M. smegmatis*. Lanes: Cell lysates of 1-Vector control *M. smegmatis*-pVV16; 2-*M.smeg*Δ*IdeR*; 3-protein marker; 4 and 5-*M.smeg*Δ*IdeR*-pVV-IdeR. (B) The growth curve of *M.smeg*Δ*IdeR* along with the corresponding vector control and mutant complemented strain. (C) The growth curve of *M.smeg*-pVV-VapC48 along with the corresponding vector control.

Table S1:

Mycobacterial proteins identified from the LC-MALDI-MS/MS of the phagosome-enriched fractions from BCG infected cells. The list corresponds to the proteins identified using MASCOT database against Mycobacterium tuberculosis complex database with more than 95% confidence ($p<0.05$).

Table S2:

Mycobacterial proteins identified from the LC-MALDI-MS/MS of the phagosome-enriched fractions from HIV-BCGinfected cells. The list corresponds to the proteins identified using MASCOT database against Mycobacterium tuberculosis complex database with more than 95% confidence ($p<0.05$).

Table S3:

Primers used in the study.

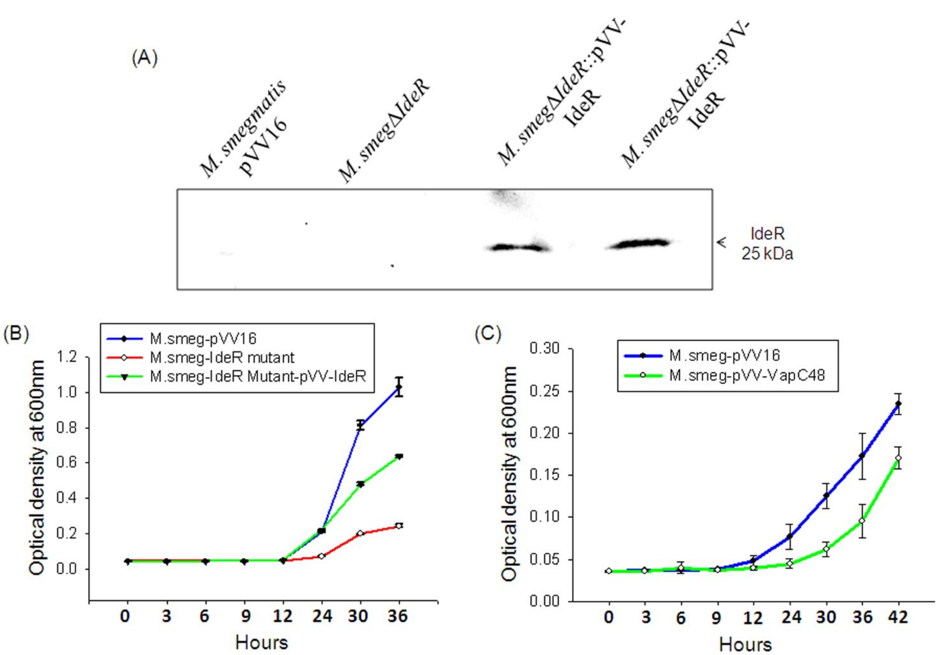

Figure S1: Western blot using anti-His antibody confirming the expression His-tagged IdeR in *M. smegmatis*. Lanes: Cell lysates of 1-Vector control *M. smegmatis*-pVV16; 2-*M.smegΔIdeR*; 3-protein marker; 4 and 5-*M.smegΔIdeR*-pVV-IdeR. (B) The growth curve of *M.smegΔIdeR* along with the corresponding vector control and mutant complemented strain. (C) The growth curve of *M.smeg*-pVV-VapC48 along with the corresponding vector control.

Table S1: Mycobacterial proteins identified from the LC-MALDI-MS/MS of the phagosome-enriched fractions from BCG infected cells. The list corresponds to the proteins identified using MASCOT database against Mycobacterium tuberculosis complex database with more than 95% confidence ($p<0.05$).

Accession	% Sequence coverage	Protein name	score	Number of Peptides matched	Observed	Mr(expt)	Mr(calc)	Delta	Miss	Score	Expect	Rank	Peptide
BCG1368	16.6	ATP synthase subunit alpha	209	6	1289.5627	1288.5554	1288.6510	-0.0956	0	2	2.5	4	R.ASEEEILTEIR.D
					1386.6976	1385.6903	1385.7085	-0.0182	1	(3)	5.1	8	K.AIDAMTPIGRGQR.Q + Deamidation (NQ)
					1553.6653	1552.6580	1552.7310	-0.0730	0	(43)	0.00024	1	R.EAYPGDVFYLHSR.L
					1607.8223	1606.8150	1606.8314	-0.0164	1	1	7.3	4	K.EVAGSLRLDLSQYR.E + Deamidation (NQ)
					1758.8632	1757.8559	1757.9423	-0.0864	1	9	0.81	2	K.RTGEVLSVPVGDGFLGR.V
					1325.6985	1324.6912	1324.7826	-0.0914	1	5	2.1	3	R.VAQEVIRILQR.Y + Deamidation (NQ)
BCG1370	9.1	ATP synthase subunit beta	125	2	1921.8884	1920.8811	1920.9581	-0.0770	0	63	3.5e-006	1	R.DEQGQDVLLFIDNIFR.F
					2741.3057	2740.2984	2740.4017	-0.1033	1	1	6.2	8	K.DLQDIIAILGIDELSEEDKQLVNR.A + 2 Deamidation (NQ)
BCG3326; BCG3362	12.5	Putative DNA glycosylase Rv3297/MT3396	36	3	1527.7728	1526.7655	1526.7940	-0.0285	0	(26)	0.017	1	R.IILEANQQEQAIR.V + 2 Deamidation (NQ)
					1684.8541	1683.8468	1683.8039	0.0430	0	(1)	7.4	3	-.MPEGDTVWHTAATLR.R
					1709.84	1708.84	1708.86	-0.0227	1	(2)	4.4	2	M.PEGDTVWHTAATLRR.H

					91	18	45						
BCG0937	2.9	Uncharacterized protein Rv0885/MT0908	30	1	1032.5363	1031.5290	1031.5222	0.0068	0	(5)	2.8	5	R.QIEIGMWR.Q
BCG3477	14.1	Antitoxin VapB47	30	1	1198.6426	1197.6353	1197.7081	-0.0727	0	(3)	3.6	7	R.ATVGLVEAIGIR.E
BCG3174	3.0	NADH-quinone oxidoreductase subunit G	25	2	1121.5306	1120.5233	1120.5183	0.0051	0	5	2.8	2	-.MTQAADTDIR.V
					1198.6378	1197.6305	1197.6539	-0.0234	0	(2)	4.9	6	R.AAELMGIQIPR.F
BCG2301c ; BCG2302c	5.0	Hypothetical protein Rv2286c/MT2344	27	1	1121.5574	1120.5501	1120.5659	-0.0157	1	27	0.012	1	R.RTNMSLLDR.W + Oxidation (M)
BCG1088	13.7	Potassium-transporting ATPase B chain	24	7	1091.5394	1090.5321	1090.5553	-0.0232	1	4	2.7	3	R.AESASAMLRR.N
					1093.4906	1092.4833	1092.5637	-0.0804	0	1	4	3	R.SLFDPMIVR.S + Oxidation (M)
					1153.5696	1152.5623	1152.6073	-0.0450	0	(1)	5.4	2	K.LNVMALHSPR.S + Oxidation (M)
					1233.6714	1232.6641	1232.6699	-0.0058	1	(13)	0.41	1	K.RSLFDPMIVR.S
					1922.8695	1921.8622	1921.9349	-0.0727	1	6	1.6	3	M.MIARMETSATAAAATSAPR.L + Oxidation (M)
					1975.9419	1974.9346	1975.0309	-0.0962	1	1	6.6	3	K.QGQTFIDRMIALVEGAAR.Q
					2049.9802	2048.9729	2048.9737	-0.0008	1	0	7.9	6	R.DEGVMSHARFVPFTAETR.M
BCG1539	20.3	Peptidoglycan endopeptidase RipA	24	7	1233.6714	1232.6641	1232.6109	0.0532	1	2	5.6	8	R.TERVNTESAAR.L
					1578.7382	1577.7309	1577.7719	-0.0409	0	(3)	3.6	1	K.TLSASSQAVMANLQR.A + 2 Deamidation (NQ)

					2406.1135	2405.1062	2405.0717	0.0345	1	0	5.9	3	K.ANQRLQDLSDEVQAEQESVNK.A + 5 Deamidation (NQ)
					847.5490	846.5417	846.4195	0.1222	0	9	0.88	1	R.VNTESAAR.L
					1210.7365	1209.7292	1209.5738	0.1555	0	1	5.1	6	K.DANAAIAAAQHR.F + 2 Deamidation (NQ)
					1605.7955	1604.7882	1604.6801	0.1081	0	2	5.9	1	R.DNAAAAEDDLEVSQR.A + 2 Deamidation (NQ)
					1804.8035	1803.7962	1803.9261	-0.1298	1	6	1.9	1	K.TLSASSQAVMANLQRAR.T + Deamidation (NQ)
BCG3940c	8.0	ESX-2 secretion system protein EccE2	23	3	1091.5328	1090.5255	1090.5883	-0.0628	1	(12)	0.39	1	K.LTGFSPRSAR.R
					1132.4844	1131.4771	1131.5520	-0.0748	0	(1)	1.9	2	R.LGSDAVAGSAQR.W + Deamidation (NQ)
					1795.8292	1794.8219	1794.9376	-0.1157	1	4	2.9	1	R.RPVKWNDPITLANNR.S + 2 Deamidation (NQ)
BCG3756c	11.0	Probable ribonuclease VapC48	36	1	1521.8158	1520.8085	1520.8133	-0.0048	0	36	0.0019	1	R.GNLVPDAHLVALMR.H + Oxidation (M)
BCG3226c	7.2	ATP-dependent DNA helicase pcrA	22	5	1048.5804	1047.5731	1047.6189	-0.0457	1	22	0.034	1	R.RLAYVGITR.A
					1247.6597	1246.6524	1246.7033	-0.0509	1	12	0.51	4	K.EIRDIVAYLR.V
					1497.7227	1496.7154	1496.7333	-0.0179	0	(0)	4.9	5	K.AIAGFVEMFDELR.G
					1944.9515	1943.9442	1944.0024	-0.0582	1	1	5.5	9	R.STQNILSAANSVIARNAGR.R + 2 Deamidation (NQ)
					2060.0139	2059.0066	2058.9857	0.0209	0	0	6.1	3	R.LDNLNELVSVAHEFSTDR.E + Deamidation (NQ)
BCG2654c	27.8	Uncharacterized protein	22	6	1627.9398	1626.9325	1626.8994	0.0331	1	4	2.1	2	R.FIYAGIADRLVHPR.E

		Rv2627c/MT2702			1654.8754	1653.8681	1653.7998	0.0684	0	1	5.8	2	R.ISQEVAAGLEFYGNR.R + Deamidation (NQ)
					1724.8271	1723.8198	1723.9364	-0.1166	0	12	0.6	1	R.MISPLSLTPLVPMPGR.F + Oxidation (M)
					1976.0194	1975.0121	1974.9224	0.0898	0	0	7.7	4	R.IFFDSGFTPHPGEPGSQR.W
					2101.0547	2100.0474	2100.0963	-0.0489	1	22	0.044	1	R.FVQAALEQSGLLDAPRTQR.D + Deamidation (NQ)
					2369.1489	2368.1416	2368.2579	-0.1163	0	10	0.79	1	R.WLEKPSGFFAQPPPLTEVAVR.K
Mb2002c	14.5	Immunogenic protein MPB64	22	2	1172.5251	1171.5178	1171.4934	0.0244	0	22	0.022	1	K.AFDWDQAYR.K + Deamidation (NQ)
					2222.0957	2221.0884	2221.0902	-0.0018	0	1	6.3	4	R.EAPYELNITSATYQSAIPPR.G + Deamidation (NQ)
BCG0489c	13.4	Putative membrane protein mmpL4	25	7	1183.6260	1182.6187	1182.5015	0.1172	0	25	0.033	1	K.EAAYYTMYR.G + Oxidation (M)
					1356.7297	1355.7224	1355.7772	-0.0548	1	3	4.1	4	K.QEIGAGLKTGIIR.S + Deamidation (NQ)
					1477.7208	1476.7135	1476.7143	-0.0008	1	1	7.6	4	R.AMKSFLSSDGHAAR.F
					1909.9784	1908.9711	1908.8808	0.0903	1	9	1	1	R.MVGDTEEMKEITEELR.D
					2214.0369	2213.0296	2213.0746	-0.0450	0	(1)	6.3	1	R.TPTVPSETQPAGRPLAMSSDR.L + Oxidation (M)
					2377.2214	2376.2141	2376.0833	0.1308	0	1	7.5	3	R.LMPQMVAQIPPQIEAMENMR.T + 2 Deamidation (NQ); 3 Oxidation (M)
					2433.1580	2432.1507	2432.0961	0.0547	1	3	3.6	1	K.AYVTGPSALAADMHHSGDRSMAR.I + 2 Oxidation (M)
BCG2361; BCG2362	5.5	Putative membrane protein mmpL9	25	5	1147.6931	1146.6858	1146.6244	0.0614	1	2	5.7	4	R.EIKATTSELR.D
					1183.62	1182.61	1182.50	0.1172	0	25	0.033	1	R.EASFYTMYR.G + Oxidation

					60	87	15						(M)
					1253.7823	1252.7750	1252.6775	0.0975	0	3	2.4	4	K.IVANSTPPEGIR.T
					1330.6431	1329.6358	1329.6347	0.0012	0	4	4.1	2	R.AVAVAPDDAQSMR.A
					1381.8073	1380.8000	1380.7725	0.0276	1	3	4	5	R.KIVANSTPPEGIR.T
BCG2953	7.2	Phenolpthiocerol synthesis polyketide synthase ppsA	51	9	1104.5413	1103.5340	1103.6008	-0.0668	0	2	4.1	4	R.ICTEITGLVR.T
					1105.5486	1104.5413	1104.5411	0.0002	0	(0)	6.4	6	M.TGSISGEADLR.H
					1187.6405	1186.6332	1186.6492	-0.0160	1	1	5.2	8	R.IDAIRALEMR.G
					1254.6670	1253.6597	1253.6840	-0.0243	1	18	0.078	1	R.RSSISQVPPQR.W
					1433.7340	1432.7267	1432.8514	-0.1246	1	2	3.6	4	R.RDGVVLAPALAPVR.G
					1536.7338	1535.7265	1535.7038	0.0227	0	10	0.75	1	R.GFDQVGALSPTGQCR.A + Deamidation (NQ)
					1558.6877	1557.6804	1557.7933	-0.1128	0	5	1.8	1	R.VLAVICGSAVNQDGR.S + Carbamidomethyl (C)
					1759.7533	1758.7460	1758.8280	-0.0819	0	2	3.4	2	R.SNGLMAPNPAAQMAVLR.A + 3 Deamidation (NQ); Oxidation (M)
					1819.8075	1818.8002	1818.7817	0.0185	0	(1)	5.1	3	R.QQGCHTMSLDWVAWR.G + 2 Deamidation (NQ)
BCG0389	14.6	Chaperone protein dnaK (Heat shock protein 70)	38	5	989.5499	988.5426	988.5263	0.0163	0	8	1	4	R.MPAVTDLVK.E + Oxidation (M)
					1567.7776	1566.7703	1566.8769	-0.1066	0	3	3.5	4	K.LLGSFELTGIPPAPR.G
					1648.7782	1647.7709	1647.8501	-0.0792	1	7	1.1	1	K.AALGGSDISAIKSAMEK.L

					1655.7609	1654.7536	1654.9002	-0.1465	1	3	2.7	7	R.QATKDAGQIAGLNVLR.I + Deamidation (NQ)
					2774.2734	2773.2661	2773.2202	0.0460	1	18	0.11	1	K.RSETFTTADDNQPSVQIQVYQGER.E + 5 Deamidation (NQ)
BCG1627	41.5	Biotin synthase	37	7	1037.4417	1036.4344	1036.5011	-0.0667	0	3	2.1	1	R.WQTLSMVR.D + Deamidation (NQ); Oxidation (M)
					1184.6724	1183.6651	1183.7189	-0.0538	1	21	0.053	1	K.AVAAFRLALPR.T
					1808.8368	1807.8295	1807.9250	-0.0955	1	3	2.9	2	K.QTAKSGATEFCIVAAVR.G + Carbamidomethyl (C)
					2259.9766	2258.9693	2258.9163	0.0531	0	0	5.8	3	R.DAGMEVCCGGILGMGETLQQR.A + Carboxymethyl (C); 2 Deamidation (NQ); 2 Oxidation (M)
					2749.2336	2748.2263	2748.2833	-0.0569	0	(1)	5.1	5	R.NEVEINIACSLGMLTAEQVDQLAAR.G + Carbamidomethyl (C); 4 Deamidation (NQ)
					2833.2883	2832.2810	2832.4729	-0.1919	1	0	5.6	10	R.QQVLQRGEGLNQDQVLAVLQLPDDR.L + Deamidation (NQ)
					3398.5046	3397.4973	3397.6345	-0.1371	1	9	0.62	2	M.TQAATRPTNDAGQDGGNNSDILVVARQQVLQR.G + 5 Deamidation (NQ)
BCG2943c	9.0	Chromosome partition protein smc	34	7	929.4736	928.4663	928.4978	-0.0314	0	1	5.5	4	R.AAAIQADLR.D + Deamidation (NQ)
					1295.6418	1294.6345	1294.7033	-0.0688	1	5	2.1	5	K.GFKSFAAPTTLR.F
					1452.7020	1451.6947	1451.7303	-0.0355	0	7	0.87	9	R.LHAAAVAAAVADCGR.L + Carbamidomethyl (C)

					1616.7947	1615.7874	1615.7914	-0.0039	1	1	5.5	1	R.AVDGASQLRDASAAQR.Q + Deamidation (NQ)
					1682.8705	1681.8632	1681.8859	-0.0227	1	(2)	3.7	8	R.QAEAAQRAAAIQADLR.D
					1773.8196	1772.8123	1772.9156	-0.1033	0	5	1.9	2	R.TQTLDDVIQLETQLR.K + Deamidation (NQ)
					2581.1060	2580.0987	2580.2853	-0.1865	1	4	2.3	1	R.EMHVIVGQGKLEEILQSRPEDR.R + 2 Deamidation (NQ); Oxidation (M)
BCG2457c	14.3	Glutamine-dependent NAD(+) synthetase	31	7	1225.6096	1224.6023	1224.5557	0.0466	1	4	2.2	4	R.LRMGTFDDNR.R + Deamidation (NQ)
					1311.5906	1310.5833	1310.6479	-0.0646	1	2	2.6	2	R.HHRELTESFR.R
					1388.6661	1387.6588	1387.6514	0.0075	1	6	1.8	6	R.QMAPGDGERGTIR.I + Deamidation (NQ)
					1494.6792	1493.6719	1493.7487	-0.0768	1	10	0.59	1	K.VSHGGALSPRGDWR.A
					1612.7744	1611.7671	1611.7780	-0.0108	0	0	6.7	5	K.VYDVTFENVQAGLR.T + 2 Deamidation (NQ)
					1682.8705	1681.8632	1681.9039	-0.0406	0	1	5.2	10	R.IDFALDPPAGDIGLLR.E
					1744.7937	1743.7864	1743.9379	-0.1515	1	8	0.76	1	R.SALPNGPKVSHGGALSPR.G
BCG0716	12.0	DNA-directed RNA polymerase beta chain	31	7	1335.6755	1334.6682	1334.6619	0.0064	1	11	0.41	2	R.GAWLEFDVDKR.D
					1542.8064	1541.7991	1541.8049	-0.0058	1	1	6.1	2	K.GETELTPEERLLR.A
					1542.8271	1541.8198	1541.8412	-0.0214	1	5	2.3	1	R.LRTVGELIQNQIR.V + 3 Deamidation (NQ)
					1692.8344	1691.8271	1691.8491	-0.0220	1	5	2.1	1	R.AGLEVRDVHPSHYGR.M

					1755.8164	1754.8091	1754.9283	-0.1192	1	1	4.5	4	R.ALMGANMQRQAVPLVR.S + Deamidation (NQ)
					2711.1184	2710.1111	2710.2906	-0.1795	1	3	2.8	4	R.QSKTAASPSPSRPQSSSNNSVPGAPNR.V + 2 Deamidation (NQ)
					3399.5056	3398.4983	3398.8271	-0.3288	1	(4)	1.7	8	K.GVIGKILPVEDMPFLADGTPVDIILNTHGVPR.R + Oxidation (M)
BCG2112c	11.7	Probable helicase helY	29	6	1184.6724	1183.6651	1183.7036	-0.0385	1	17	0.14	2	R.SVVGLVRGIER.G
					1380.5898	1379.5825	1379.6906	-0.1080	1	4	2.2	3	R.ADEQAHRITPSR.E
					1448.7189	1447.7116	1447.7506	-0.0390	0	2	4.2	3	R.GLAAHHAGMLPAFR.H
					1701.8450	1700.8377	1700.8668	-0.0291	1	(3)	2.9	1	R.WCRQVLDLLDQVR.N + Carbamidomethyl (C); Deamidation (NQ)
					1719.8022	1718.7949	1718.8079	-0.0130	1	1	4.5	10	R.AGCDAAVTQCLRSPLR.L + Carboxymethyl (C); Deamidation (NQ)
					2996.3206	2995.3133	2995.4782	-0.1649	1	4	2.1	3	K.LDAEGLLPAITFVFSRAGCDAAVTQCLR.S + Carboxymethyl (C); Deamidation (NQ)
BCG1085c	14.5	Sensor protein kdpD	28	7	1419.6412	1418.6339	1418.7088	-0.0749	1	6	1.6	1	R.YAPDCVVRVNAGR.V
					1567.7776	1566.7703	1566.8803	-0.1100	1	13	0.34	1	K.TAKLLEGIEMIPPR.Y
					1571.8406	1570.8333	1570.8467	-0.0133	1	1	4.4	5	R.GARFPELDVEAVLR.R
					1755.8164	1754.8091	1754.9712	-0.1621	1	5	1.8	1	R.IASKSSAELMVVHVIR.G + Oxidation (M)
					1762.89	1761.89	1761.97	-0.0836	1	0	4.7	2	R.ERVLINVIDEGPGVPR.G

					73	00	36						
					1932.1108	1931.1035	1931.0475	0.0560	1	2	4.1	2	R.RASQEAELLALFAGSVLR.G + Deamidation (NQ)
					2005.9008	2004.8935	2004.9469	-0.0533	1	2	4	7	R.VRQGASTGETVACVGTNPCR.D
BCG0717	5.1	DNA-directed RNA polymerase beta' chain	27	4	944.4952	943.4879	943.5450	-0.0571	1	15	0.23	3	R.KGLADTALR.T
					1314.6951	1313.6878	1313.6826	0.0052	0	6	1.4	3	K.VQAAIINDLAER.Y + 2 Deamidation (NQ)
					1819.8251	1818.8178	1818.9264	-0.1086	0	1	4.9	4	R.EGLTVLEYFINTHGAR.K
					2355.0615	2354.0542	2354.2449	-0.1907	0	5	1.9	4	R.ILMLSSNNILSPASGRPLAMPR.L + Deamidation (NQ); Oxidation (M)
BCG3215c	6.0	Hypothetical UPF0182 protein Mb3215c	26	4	1349.6907	1348.6834	1348.6734	0.0100	1	12	0.34	1	R.NGNLRDYVVAAR.E + 2 Deamidation (NQ)
					1384.6375	1383.6302	1383.6782	-0.0480	1	13	0.32	1	K.KGDFAAYGSALQR.L + Deamidation (NQ)
					1481.8254	1480.8181	1480.8361	-0.0180	0	0	5.1	5	K.VAALQEIQAAIGAAR.D
					1483.6919	1482.6846	1482.7426	-0.0580	1	2	3.6	1	R.ELNPDRLIDNQR.D + Deamidation (NQ)
BCG1357	11.0	Transcription termination factor rho homolog	25	5	1305.5776	1304.5703	1304.6095	-0.0392	0	13	0.27	1	R.LDSINGGSVEDAK.K + Deamidation (NQ)
					1431.6693	1430.6620	1430.7616	-0.0996	0	6	1.5	3	K.TTILQDIANAITR.N + 2 Deamidation (NQ)
					1515.7021	1514.6948	1514.7841	-0.0892	1	4	3.1	4	R.RVFPAVDVNPSGTR.K + Deamidation (NQ)
					1685.8181	1684.8108	1684.9471	-0.1363	1	1	5.5	5	K.AGKTTILQDIANAITR.N
					1815.9473	1814.9400	1814.9791	-0.0391	1	2	4.5	2	K.RPEFGKLTPLYPNQR.L

BCG1194c	7.2	5-methyltetrahydropteroyltriglutamate--homocysteine methyltransferase	25	4	1121.5673	1120.5600	1120.5625	-0.0025	1	7	1.1	1	R.RADQAEYLR.W
					1431.6693	1430.6620	1430.7630	-0.1010	1	3	2.5	6	R.RQPFTATITGSPR.I
					1433.7340	1432.7267	1432.7674	-0.0406	0	12	0.38	1	R.LGLDVLVHGEPER.N
					1460.6379	1459.6306	1459.7129	-0.0823	1	3	2.7	6	R.LWVNPDCGLKTR.N + Carboxymethyl (C); Deamidation (NQ)
BCG0453c	22.1	Probable thiamine-phosphate pyrophosphorylase	25	3	958.5460	957.5387	957.4879	0.0508	1	6	1.8	2	R.DKGSPGELR.F
					1452.7020	1451.6947	1451.7554	-0.0607	1	13	0.24	2	R.LASARLYLCTDAR.R
					2130.0793	2129.0720	2129.1003	-0.0283	0	7	1	2	R.GDLAQFAEAALAGGVDIIQLR.D + 2 Deamidation (NQ)
BCG2367c	20.0	Deoxyguanosine triphosphate triphosphohydrolase-like protein	24	4	1184.6724	1183.6651	1183.6560	0.0091	1	14	0.28	4	R.LADKTQVVGPR.E + Deamidation (NQ)
					1513.7422	1512.7349	1512.8259	-0.0910	0	0	6	4	K.VVDAQGLSAGLNLTR.A
					2130.0793	2129.0720	2129.1918	-0.1197	1	8	0.76	1	R.AEVAVLKILALQFIMSDPR.H + Oxidation (M)
					3398.5046	3397.4973	3397.6182	-0.1209	0	2	2.7	7	R.GMAIGLGCDLDLVELAGLAHDIGHPPYGHNGER.A + Deamidation (NQ)
BCG1182	8.4	Probable glucose-6-phosphate 1-dehydrogenase	24	3	985.4664	984.4591	984.4698	-0.0107	0	4	1.7	3	R.AMAPLDPDR.C
					1254.6670	1253.6597	1253.7244	-0.0647	1	18	0.078	1	R.RVPALAFLPNR.R + Deamidation (NQ)
					1372.6959	1371.6886	1371.6782	0.0104	0	2	4.6	6	R.GSWGPEAAQSLLR.G + Deamidation (NQ)
Mb2007c	17.2	Putative HTH-type	24	3	1452.7020	1451.6947	1451.7481	-0.0533	1	13	0.23	1	R.IEDQDHSARLLR.E

		transcriptional regulator Mb2007c			1682.8705	1681.8632	1681.9297	-0.0665	1	9	0.72	2	R.EKPCRATTAGIPLLR.L + Carbamidomethyl (C)
					2017.9869	2016.9796	2016.9356	0.0440	1	4	2.5	1	R.EGVAMGAVTTERNPVPGCR.V + Carbamidomethyl (C); Deamidation (NQ); Oxidation (M)
BCG1573	11.1	Putative fatty-acid--CoA ligase fadD25	24	4	1138.5665	1137.5592	1137.5600	-0.0008	1	14	0.17	1	R.RAQCVELYR.Q + Deamidation (NQ)
					1389.6616	1388.6543	1388.7333	-0.0790	0	4	2.8	1	-.MSVVESSLPGVLR.E + Oxidation (M)
					2056.9133	2055.9060	2056.0298	-0.1238	1	3	3.7	1	R.TGDSGFLSEGELFIMGRIK.D
					2126.0117	2125.0044	2125.0228	-0.0184	1	(1)	5.6	3	R.SWDGVEETLTWSQLYRR.T
BCG0932	49.7	Putative HTH-type transcriptional regulator Mb0904	23	4	943.5424	942.5351	942.5069	0.0282	0	10	0.69	2	R.VRPPSMTR.V
					1175.5951	1174.5878	1174.6380	-0.0501	0	3	3.1	2	R.LASDLSLAVMR.L
					1600.7428	1599.7355	1599.8369	-0.1013	1	5	1.7	1	R.QEWLAERLATLNR.S + Deamidation (NQ)
					3084.3748	3083.3675	3083.5808	-0.2133	0	5	1.4	4	R.NPSSPVSLSQLSALTTLANEGAMTPGALAIR.E + Deamidation (NQ); Oxidation (M)
BCG1389c	3.3	Alpha-1,4-glucan:maltose-1-phosphate maltosyltransferase	29	2	1570.8674		1570.9427	-0.0753	0	15		1	SLQPFITRLNIIR
					2024.0833		2024.0083	0.075	0	15		1	DFASALDQGRSLQPFITR + Deamidation (NQ)
BCG2562c	9.2	Chorismate synthase	30	2	1712.9463		1712.886	0.0603	0	17		1	AGGLEGGMTNGQPLRVR
					2172.1531		2172.0278	0.1253	0	13		1	WETVMAADPVDPAELADVAR + Oxidation (M)

BCG1550	10.0	Uncharacterized protein Mb1524	40	3	958.4602		958.4952	-0.035	0	15		1	DQINAQLR + Deamidation (NQ)
					1574.8329		1574.8571	-0.0242		13		5	AMILTAEGTREAAIK
					2561.1716		2561.2988	-0.1272	0	13		1	NVVGGMTLEQTLTSRDQINAQLR + Deamidation (NQ) + Oxidation (M)
BCG0788	12.1	Uncharacterized protein Rv0738/MT0763	32	2	1361.6738		1361.7059	-0.0321	0	12		1	AQDAFAALLANVR + Deamidation (NQ)
					2441.1016		2441.2063	-0.1047	0	20		1	MDPLMAHQRAQDAFAALLANVR + Deamidation (NQ)
BCG0946	9.7	Uncharacterized protein Mb0918	32	2	2126.0139		2126.1604	-0.1465	0	13		1	VPPLSLTNDAIELFVDRAR
					2131.0024		2131.1406	-0.1382	0	19		1	SRLALQIGAQIAHEFTYGR

Table S2: Mycobacterial proteins identified from the LC-MALDI-MS/MS of the phagosome-enriched fractions from HIV-BCG infected cells. The list corresponds to the proteins identified using MASCOT database against Mycobacterium tuberculosis complex database with more than 95% confidence (p<0.05).

Accession	% Sequence coverage	Protein name	score	Number of Peptides matched	Observed	Mr(expt)	Mr(calc)	Delta	Miss	Score	Expect	Rank	Peptide
BCG1368	24.6	ATP synthase subunit alpha	200	9	1044.5963	1043.5890	1043.5433	0.0457	0	2	6.2	4	K.AIDAMTPIGR.G
					1289.5624	1288.5551	1288.6510	-0.0959	0	3	2	6	R.ASEEEILTEIR.D
					1927.9583	1926.9510	1927.0084	-0.0574	1	(0)	6.8	7	K.EPLQTGIKAIDAMTPIGR.G + Deamidation (NQ); Oxidation (M)
					901.5200	900.5127	900.4777	0.0350	1	5	2.4	3	K.AQLERGAR.L + Deamidation (NQ)
					3575.6421	3574.6348	3574.7612	-0.1264	0	0	4.1	1	K.QPQSQPMPVEEQVVSIFLGTGGHLDSVPVEDVR.R + Deamidation (NQ)
					1306.6006	1305.5933	1305.6023	-0.0090	0	1	5.9	5	R.FETELLDHMR.A + Oxidation (M)
					1334.7050	1333.6977	1333.6361	0.0616	1	0	6.7	4	R.DSQKLTEEAADK.L
					1553.6958	1552.6885	1552.7310	-0.0425	0	91	5e-009	1	R.EAYPGDVFYLHSR.L
					1607.7731	1606.7658	1606.8314	-0.0656	1	6	1.9	1	K.EVAGSLRLDLSQYR.E + Deamidation (NQ)

BCG1370	23.7	ATP synthase subunit beta	88	6	1325.7152	1324.7079	1324.7826	-0.0747	1	(1)	3.9	3	R.VAQEVIRILQR.Y + Deamidation (NQ)
					1779.0247	1778.0174	1777.9686	0.0488	0	0	2.4	1	K.TLTLEVAQHLGDNLVR.T
					1800.8171	1799.8098	1799.8941	-0.0842	0	(3)	3.1	7	R.EGNDLWVELAEANVLK.D + Deamidation (NQ)
					1921.8965	1920.8892	1920.9581	-0.0689	0	(46)	0.00016	1	R.DEQGQDVLLFIDNIFR.F
					1998.0237	1997.0164	1997.0792	-0.0628	1	1	7.2	1	R.GVEVIDTGRSISVPVGEGVK.G
					2741.2742	2740.2669	2740.4017	-0.1348	1	(2)	4.4	7	K.DLQDIIAILGIDELSEEDKQLVNR.A + 2 Deamidation (NQ)
BCG1539	12.7	Peptidoglycan endopeptidase RipA	34	3	1233.6842	1232.6769	1232.6109	0.0660	1	4	2.8	8	R.TERVNTESAAR.L
					1594.8560	1593.8487	1593.7668	0.0819	0	(26)	0.022	1	K.TLSASSQAVMANLQR.A + 2 Deamidation (NQ); Oxidation (M)
					2827.2883	2826.2810	2826.3295	-0.0485	1	3	2.9	10	R.LVAWSSEGGQGAPPFRMWDPGSGPAGGR.A
BCG2301c; BCG2302c	5.0	Uncharacterized protein Rv2286c/MT2344	31	1	1121.5463	1120.5390	1120.5659	-0.0268	1	(21)	0.059	1	R.RTNMSLLDR.W + Oxidation (M)
BCG0717	6.0	DNA-directed	29	6	842.4922	841.4849	841.4657	0.0192	0	1	6.5	4	R.APDGTLIR.D

		RNA polymerase subunit beta'			944.4871	943.4798	943.5450	-0.0652	1	(16)	0.3	2	R.KGLADTALR.T
					1314.7145	1313.7072	1313.6826	0.0246	0	(20)	0.066	1	K.VQAAIINDLAER.Y + 2 Deamidation (NQ)
					1331.6796	1330.6723	1330.7357	-0.0633	1	0	8	4	K.LIPAGTGINRYR.N + Deamidation (NQ)
					1409.7603	1408.7530	1408.7972	-0.0442	1	(0)	5.7	6	K.HIEVIVRQMLR.R + Oxidation (M)
					1750.9490	1749.9417	1749.8468	0.0950	1	(2)	4.4	4	K.RMLQESVDALFDNGR.R
BCG1550	13.4	Uncharacterized protein Rv1488/MT1533.2	27	3	958.4566	957.4493	957.4879	-0.0386	0	(9)	0.86	1	R.DQINAQLR.G + Deamidation (NQ)
					1574.8308	1573.8235	1573.8497	-0.0262	1	(17)	0.14	2	R.AMILTAEGTREAAIK.Q
					2569.2158	2568.2085	2568.2352	-0.0267	0	1	5.3	1	K.AGRPTPEMLAYQYLQTLPEMAR.G + Deamidation (NQ); 2 Oxidation (M)
BCG0123	14.5	Uncharacterized protein Rv0090/MT0099	24	2	944.4871	943.4798	943.5087	-0.0288	0	(15)	0.34	3	R.GAIQATLDR.D
					2557.2480	2556.2407	2556.2430	-0.0023	0	3	2.9	3	R.WELITCGLGGHVTYAPDDAALAAR.L
BCG1689	4.0	Phenylalanine--tRNA ligase beta subunit	23	2	1361.6910	1360.6837	1360.7462	-0.0625	0	6	2.1	5	R.QPQHVAAVLAGLR.E + 2 Deamidation (NQ)
					1799.8248	1798.8175	1798.8883	-0.0708	0	23	0.03	1	R.RPTDDEIAMLDASLPR.Q

BCG3932	20.4	ESX-1 secretion system protein eccB1	22	4	1361.6910	1360.6837	1360.7615	-0.0778	1	22	0.044	1	K.VQVSGWRFLLR.R + Deamidation (NQ)
					2834.2769	2833.2696	2833.3268	-0.0572	1	0	4.6	8	K.FVALQSPDPRYTESMYYIDPQGVR.Y + 2 Deamidation (NQ)
					2839.3567	2838.3494	2838.5127	-0.1632	1	2	5.5	2	R.LLVDGPVLSKDAALLEHDTLPADPSPR.K
					2937.3179	2936.3106	2936.4887	-0.1781	1	0	4.9	1	R.HLPISPSAMNMGIKQIHGTATVYLDGGK.F + Deamidation (NQ)
BCG3331c	5.6	Glycerol-3-phosphate dehydrogenase 2	22	2	958.5484	957.5411	957.5719	-0.0308	1	22	0.048	1	K.RSSLIGGIR.Y
					2172.1665	2171.1592	2171.1633	-0.0040	1	1	4.8	2	R.TAAHYGAVVRCSTQVVALLR.E
BCG0626	15.5	Antitoxin VapB26	22	1	958.4647	957.4574	957.5243	-0.0669	0	(13)	0.38	1	R.GVSEAQVIR.E
BCG2953	12.9	Phenolpthiocerol synthesis polyketide synthase ppsA	35	14	860.5117	859.5044	859.4512	0.0533	1	5	2.3	2	K.RLTDAQR.D + Deamidation (NQ)
					1105.5631	1104.5558	1104.5411	0.0147	0	1	7.1	2	M.TGSISGEADLR.H
					1254.6572	1253.6499	1253.6840	-0.0341	1	5	2	4	R.RSSISQVPPQR.W
					1409.7603	1408.7530	1408.7211	0.0319	0	(1)	5.1	5	R.LAPDAKPYQGGHR.F
					1743.7585	1742.7512	1742.8331	-0.0818	0	(18)	0.065	1	R.SNGLMAPNPAAQMAVLR.A + 3 Deamidation (NQ)
					2348.0769	2347.0696	2347.0788	-0.0092	0	0	5	2	R.HVTAQLSSSPSDSASSLNEHHR.A + Deamidation (NQ)

					2619.2729	2618.2656	2618.3308	-0.0652	1	8	1.3	1	K.ELDTDRPFAELGLNSLMAMAIRR.E
					1104.6173	1103.6100	1103.6008	0.0092	0	12	0.41	1	R.ICTEITGLVR.T
					1187.6797	1186.6724	1186.6492	0.0232	1	8	1.1	3	R.IDAIRALEMR.G
					1536.8268	1535.8195	1535.7038	0.1157	0	10	0.65	1	R.GFDQVGALSPTGQCR.A + Deamidation (NQ)
					1558.7152	1557.7079	1557.7933	-0.0853	0	2	4.2	1	R.VLAVICGSAVNQDGR.S + Carbamidomethyl (C)
					1661.8401	1660.8328	1660.8056	0.0272	1	1	5.1	3	R.RDWQLDTLDTELR.R + Deamidation (NQ)
					1819.8851	1818.8778	1818.7817	0.0961	0	3	3.3	1	R.QQGCHTMSLDWVAWR.G + 2 Deamidation (NQ)
					3329.4670	3328.4597	3328.5716	-0.1119	0	1	4.2	2	R.VSATAGMLADWMEGPGADVALADVAHTLNHHR.S + Oxidation (M)
BCG0332	15.1	ESX-3 secretion system protein EccE3	22	3	1494.7448	1493.7375	1493.7474	-0.0099	0	0	6.3	4	R.LDGHTDAPADLLTR.L
					1567.7195	1566.7122	1566.8253	-0.1131	0	(0)	5.6	3	R.VSANAELPDTLPAIR.S + Deamidation (NQ)
					1611.7700	1610.7627	1610.8740	-0.1112	1	22	0.036	1	R.SARIPLQETAQVAAR.R + Deamidation (NQ)
BCG2858c	10.4	Ribosome-binding factor A	21	1	1595.7430	1594.7357	1594.8063	-0.0705	0	(16)	0.17	1	R.DNGSVAQSPAPGGLGIR.T
BCG2943	14.3	Chromoso	41	11	929.5039	928.4966	928.4978	-0.0011	0	1	6	7	R.AAAIQADLR.D + Deamidation (NQ)

c		me partition protein smc											
					935.6146	934.6073	934.5824	0.0249	1	12	0.15	1	R.LLAGRLHR.A
					1198.7412	1197.7339	1197.6829	0.0510	1	1	3.7	6	R.GVEVEARLAVR.T
					1215.6896	1214.6823	1214.6553	0.0270	1	13	0.27	1	R.QQRLAAMAAVR.D + Deamidation (NQ)
					1389.7786	1388.7713	1388.6830	0.0883	1	5	2.6	1	R.IEDAAMRAQQTR.A
					1623.7531	1622.7458	1622.7536	-0.0077	1	0	4.9	3	R.AQQTRAEFETVQGR.I + 3 Deamidation (NQ)
					2581.2058	2580.1985	2580.2853	-0.0867	1	0	6.1	1	R.EMHVIVGQGKLEEILQSRPEDR.R + 2 Deamidation (NQ); Oxidation (M)
					2883.3523	2882.3450	2882.3239	0.0211	0	2	3.6	1	R.QDAAEQALAALNESDTAISAMYEQLGR.L + Deamidation (NQ); Oxidation (M)
					1452.7020	1451.6947	1451.7303	-0.0355	0	7	0.86	5	R.LHAAAVAAAVADCGR.L
					1616.7947	1615.7874	1615.7914	-0.0039	1	1	5.8	1	R.AVDGASQLRDASAAQR.Q + Deamidation (NQ)
					1684.8646	1683.8573	1683.8539	0.0034	1	11	0.73	2	R.QAEAAQRAAAIQADLR.D + 2 Deamidation (NQ)
BCG2962c	6.2	Mycocerosic acid synthase	35	9	972.5369	971.5296	971.5036	0.0261	0	10	0.81	3	R.ANLEQAGLR.G + Deamidation (NQ)
					1046.5957	1045.5884	1045.5668	0.0216	0	1	5.4	6	R.EQPLPHGLR.G

					1168.6960	1167.6887	1167.6070	0.0817	0	5	1.6	3	R.VISSEHPMLR.T
					1187.6797	1186.6724	1186.6094	0.0630	1	10	0.58	2	R.DLIARWEQR.D + Deamidation (NQ)
					1287.6378	1286.6305	1286.7094	-0.0789	1	2	3.3	5	R.GRDGVVIVYGPR.V
					1428.6964	1427.6891	1427.8030	-0.1139	1	3	3.1	1	K.LAAAGCGRIVLTAR.S + Carbamidomethyl (C)
					1586.8013	1585.7940	1585.8576	-0.0636	1	0	5.5	7	R.RSVAVTPEQAPLYR.R
					1699.9963	1698.9890	1698.9417	0.0474	1	6	1.5	1	R.LVSERLLTLGWQQR.A + Deamidation (NQ)
					2145.1021	2144.0948	2143.9691	0.1257	0	2	3.9	6	R.AAGADIVVECGNIAEPDTADR.L + Carboxymethyl (C)
BCG3661c	10.1	Probable ATP-dependent Clp protease ATP-binding subunit	34	6	957.5175	956.5102	956.6018	-0.0916	1	4	2.4	5	K.KVLELSLR.E
					1056.5713	1055.5640	1055.5546	0.0094	1	7	1	5	R.RMTAPPDLR.E
					1199.6920	1198.6847	1198.6557	0.0291	0	1	5.5	5	R.EGEGVAAQVLVK.L
					1362.7444	1361.7371	1361.7224	0.0147	1	2	3.2	2	K.SKDMALVLTDAAK.A
					1523.7928	1522.7855	1522.8354	-0.0499	1	18	0.1	1	R.TKNNPVLIGEPGVGK.T + Deamidation (NQ)
					1691.8396	1690.8323	1690.8559	-0.0236	0	1	4.2	1	R.VSITDAAMVAAATLADR.Y + Oxidation (M)

BCG1655	7.0	Pyruvate kinase	24	2	1301.7341	1300.7268	1300.5830	0.1439	0	22	0.033	1	R.ITVGACEGSHDR.V + Carbamidomethyl (C)
					1792.9335	1791.9262	1791.9366	-0.0104	1	2	4.2	1	R.LDAKALVAFTQSGDTVR.R + Deamidation (NQ)
BCG1633	8.3	L-aspartate oxidase	24	3	1015.5195	1014.5122	1014.6185	-0.1063	1	2	4	2	R.AGRSVVVLSK.A
					1273.7686	1272.7613	1272.6496	0.1117	0	11	0.48	1	R.ALQDAAGMLDIR.T
					1678.8776	1677.8703	1677.7225	0.1478	0	11	0.4	1	R.GADDANAVCVQALVAVC.- + Carboxymethyl (C); 2 Deamidation (NQ)
BCG3299	7.4	Probable cation-transporting P-type ATPase C	24	4	957.5175	956.5102	956.5767	-0.0665	1	4	2.7	7	R.RAISELLR.G
					1046.5957	1045.5884	1045.4941	0.0943	0	1	5.1	5	R.GNQDTAWVR.L
					1368.7236	1367.7163	1367.8322	-0.1159	0	18	0.086	1	R.MVIGGVALALLGVR.R
					1579.8623	1578.8550	1578.8114	0.0437	1	1	5.2	9	R.APHSAEIRNTDVLR.M + Deamidation (NQ)
BCG0946	13.0	Hypothetical protein Mb0918	23	3	843.5034	842.4961	842.4133	0.0828	0	3	3.2	7	K.QPNQVVR.A + 3 Deamidation (NQ)
					2126.0994	2125.0921	2125.1531	-0.0610	1	11	0.5	2	R.VPPLSLTNDAIELFVDRAR.R
					2131.1558	2130.1485	2130.1333	0.0152	1	9	0.52	1	K.SRLALQIGAQIAHEFTYGR.W

BCG2112c	15.0	Probable helicase helY	23	9	1199.6920	1198.6847	1198.6557	0.0290	0	8	1.1	1	R.QVLDLLDQVR.N + Deamidation (NQ)
					1380.6587	1379.6514	1379.6906	-0.0391	1	5	1.5	2	R.ADEQAHRITPSR.E
					1575.9177	1574.9104	1574.8740	0.0364	1	2	3	1	R.QALTQTSRLSTTLR.A
					1701.9652	1700.9579	1700.8668	0.0911	1	4	2.6	1	R.WCRQVLDLLDQVR.N + Carbamidomethyl (C); Deamidation (NQ)
					2162.1794	2161.1721	2161.1491	0.0231	1	2	3.7	4	R.QVLDLLDQVRNAAPNPELR.A + Deamidation (NQ)
					2257.1917	2256.1844	2256.1386	0.0459	1	0	5.1	2	R.EFIDGPATDPVVTDDGRLLAR.I
					2995.4519	2994.4446	2994.4942	-0.0496	1	1	4.5	1	K.LDAEGLLPAITFVFSRAGCDAAVTQCLR.S + Carbamidomethyl (C); Deamidation (NQ)
					1184.6724	1183.6651	1183.7036	-0.0385	1	17	0.14	2	R.SVVGLVRGIER.G
					1448.7189	1447.7116	1447.7506	-0.0390	0	2	4.6	2	R.GLAAHHAGMLPAFR.H
BCG0716	8.4	DNA-directed RNA polymerase beta chain	22	7	1031.5930	1030.5857	1030.5407	0.0450	1	7	1.2	1	K.ISDGDKLAGR.H
					1133.6176	1132.6103	1132.5546	0.0557	0	0	5.6	5	M.LEGCILADSR.Q + Carbamidomethyl (C)
					1335.7358	1334.7285	1334.6619	0.0667	1	12	0.32	2	R.GAWLEFDVDKR.D

					1388.7799	1387.7726	1387.7095	0.0631	0	2	4.1	5	K.ALGWTSEQIVER.F
					1542.8064	1541.7991	1541.8049	-0.0058	1	3	3.2	2	K.GETELTPEERLLR.A
					1542.8271	1541.8198	1541.8412	-0.0214	1	5	2.1	1	R.LRTVGELIQNQIR.V + 3 Deamidation (NQ)
					1692.8344	1691.8271	1691.8491	-0.0220	1	5	2.1	1	R.AGLEVRDVHPSHYGR.M
BCG0123	4.3	Uncharacterized protein Mb0093	22	1	944.5690	943.5617	943.5087	0.0531	0	22	0.053	1	R.GAIQATLDR.D
BCG2433c	22.9	Hypothetical UPF0230 protein Mb2440c	21	4	1349.7496	1348.7423	1348.6921	0.0503	0	3	2.9	4	R.SAAMGVGFAALAAGR.A
					1368.7236	1367.7163	1367.7408	-0.0245	0	8	0.89	3	R.AAELTAAELGPAVR.V
					1508.6791	1507.6718	1507.7267	-0.0548	1	7	1.3	2	R.DGVDEIPDDIHKR.H
					1601.9058	1600.8985	1600.9300	-0.0315	0	4	2.3	3	R.QVPLHILLDGLDLR.D
BCG1872c	8.9	Malate synthase G	23	4	1199.6920	1198.6847	1198.5982	0.0865	0	2	5.1	4	K.ELAWAPDEIR.E
					1268.7445	1267.7372	1267.6633	0.0740	1	5	1.6	1	K.ARAAEKPAPSDR.A
					2763.3274	2762.3201	2762.4562	-0.1361	1	2	3.7	4	K.VVADLTPQNQALLNARDELQAQIDK.W

					1301.7008	1300.6935	1300.7139	-0.0204	0	23	0.036	1	R.ISSQLLANWLR.H + Deamidation (NQ)
BCG3470	15.3	Hypothetical glycosyl hydrolase Rv3401/MT3509	21	2	1599.7400	1598.7327	1598.8926	-0.1599	1	4	3.1	1	R.TTVICGLRPGQKLR.I + Carbamidomethyl (C); Deamidation (NQ)
					2333.1335	2332.1262	2332.1467	-0.0205	0	18	0.066	1	R.IVTGDGSLEEECGLAVLIETAR.L + Carboxymethyl (C)
BCG0535c	3.0	Hypothetical protein Rv0493c/MT0513	21	1	956.4260	955.4187	955.5352	-0.1164	1	21	0.039	1	R.SAALPRWR.G
BCG1479	10.1	Putative lipoprotein lprH precursor	20	2	989.5793	988.5720	988.4695	0.1026	0	16	0.17	1	M.ACLGRPGCR.G + Carbamidomethyl (C)
					1091.6211	1090.6138	1090.5407	0.0731	0	5	1.8	1	R.APFGQVTQSR.V + Deamidation (NQ)
BCG0447	8.3	Phosphate acetyltransferase	20	4	901.5200	900.5127	900.5141	-0.0013	1	2	4.2	9	K.SAGRLLQR.G + Deamidation (NQ)
					929.5039	928.4966	928.4978	-0.0011	0	9	1	2	R.KPVNDLSR.G + Deamidation (NQ)
					1600.8143	1599.8070	1599.9208	-0.1138	1	5	1.8	1	K.VGVFRPITRLSAER.D
					1877.8734	1876.8661	1877.0846	-0.2185	1	4	2.6	1	K.AVQRSAGAIAIGPVLQGLR.K + Deamidation (NQ)
BCG2596	17.9	Putative 2-dehydropantoate 2-reductase	25	3	1307.7061	1306.6988	1306.6815	0.0173	1	3	4.1	1	R.RYVAECLAVAR.A
					1363.6803	1362.6730	1362.7156	-0.0426	0	25	0.021	1	R.AAHRPLEWDLR.N

					1733.9165	1732.9092	1732.9657	-0.0565	1	1	6.6	3	K.LLVNALAGFMVLSGRR.S + Deamidation (NQ); Oxidation (M)
BCG2216c	9.9	Cytochrome c oxidase subunit 2	24	3	927.4753	926.4680	926.5045	-0.0365	1	24	0.016	1	R.GSGGPARGLR.Q
					1024.4705	1023.4632	1023.5349	-0.0716	1	2	2.1	1	R.RGELAPQPVG.- + Deamidation (NQ)
					1187.6346	1186.6273	1186.6306	-0.0032	1	0	7.8	9	R.IDGKTNAEALR.A
BCG0006; BCG0036	6.3	DNA gyrase subunit A	22	4	941.5762	940.5689	940.5705	-0.0016	0	22	0.024	1	R.IAQVIQIR.G + Deamidation (NQ)
					985.5170	984.5097	984.5716	-0.0618	0	15	0.18	2	R.SGGIVAVNLR.D
					1419.6454	1418.6381	1418.7153	-0.0772	1	2	3.6	2	K.RTAIEEYPVQGR.G + Deamidation (NQ)
					1587.7296	1586.7223	1586.8337	-0.1114	0	2	4.3	5	R.LMNLGEGDTLLAIAR.N + Deamidation (NQ)
BCG2562c	10.7	Chorismate synthase	20	3	1030.6160	1029.6087	1029.5818	0.0269	0	20	0.084	1	R.DAVTVLSGIR.H
					1249.6021	1248.5948	1248.6098	-0.0150	0	3	4.2	5	K.GVEIGDGFQTAR.R
					1457.7482	1456.7409	1456.7092	0.0317	0	3	4.4	3	R.AGGLEGGMTNGQPLR.V
BCG1085c	3.6	Sensor protein kdpD	23	2	1419.6804	1418.6731	1418.7088	-0.0357	1	4	2.2	1	R.YAPDCVVRVNAGR.V
					1567.8699	1566.8626	1566.8803	-0.0177	1	18	0.1	1	K.TAKLLEGIEMIPPR.Y

BCG0389	12.2	Chaperone protein dnaK (Heat shock protein 70)	21	4	1567.8699	1566.8626	1566.8769	-0.0143	0	4	2.6	4	K.LLGSFELTGIPPAPR.G
					1604.8267	1603.8194	1603.8318	-0.0124	1	0	6.7	6	R.AEFQRITQDLLDR.T
					1655.8259	1654.8186	1654.9002	-0.0815	1	4	2.1	1	R.QATKDAGQIAGLNVLR.I + Deamidation (NQ)
					2774.2751	2773.2678	2773.2202	0.0477	1	12	0.39	1	K.RSETFTTADDNQPSVQIQVYQGER.E + 5 Deamidation (NQ)
BCG1627	21.2	Biotin synthase	31	3	1184.6724	1183.6651	1183.7189	-0.0538	1	21	0.052	1	K.AVAAFRLALPR.T
					2749.2336	2748.2263	2748.2833	-0.0569	0	(1)	2.8	1	R.NEVEINIACSLGMLTAEQVDQLAAR.G + 4 Deamidation (NQ)
					3398.5046	3397.4973	3397.6345	-0.1371	1	9	0.3	1	M.TQAATRPTNDAGQDGGNNSDILVVARQQVLQR.G + 5 Deamidation (NQ)
BCG3633c	4.0	Iron-dependent extradiol dioxygenase	27	1	1046.5651	1045.5578	1045.5556	0.0022	0	27	0.0092	1	K.GAPEGALYLR.M
BCG3215c	7.6	Hypothetical UPF0182 protein Mb3215c	27	5	1349.6907	1348.6834	1348.6734	0.0100	1	12	0.29	1	R.NGNLRDYVVAAR.E + 2 Deamidation (NQ)
					1384.6375	1383.6302	1383.6782	-0.0480	1	12	0.25	1	K.KGDFAAYGSALQR.L + Deamidation (NQ)
					1428.6558	1427.6485	1427.6932	-0.0447	0	0	3.6	4	R.NFLFSNVIGSNSK.I + 2 Deamidation (NQ)
					1481.825	1480.818	1480.836	-0.0180	0	0	3.8	5	K.VAALQEIQAAIGAAR.D

					4	1	1							
					1483.6919	1482.6846	1482.7426	-0.0580	1	2	2.5	1	R.ELNPDRLIDNQR.D + Deamidation (NQ)
Mb2007c	17.2	Putative HTH-type transcriptional regulator Mb2007c	26	3	1452.7020	1451.6947	1451.7481	-0.0533	1	13	0.23	1	R.IEDQDHSARLLR.E
					1682.8820	1681.8747	1681.9297	-0.0550	1	24	0.025	1	R.EKPCRATTAGIPLLR.L + Carbamidomethyl (C)
					2017.9869	2016.9796	2016.9356	0.0440	1	4	2.5	1	R.EGVAMGAVTTERNPVPGCR.V + Deamidation (NQ); Oxidation (M)
BCG2636c	5.2	Phosphatidylinositol mannoside acyltransferase	23	3	1363.6094	1362.6021	1362.6383	-0.0362	1	13	0.11	1	R.NNRVVCLMAER.D + 2 Deamidation (NQ)
					1389.6616	1388.6543	1388.6472	0.0071	1	6	1.7	1	R.NAFDTGARYFAR.H + Deamidation (NQ)
					2199.9509	2198.9436	2199.0531	-0.1094	1	4	1.1	1	R.SSVTRTATDWAYAAGWMAVR.A
BCG2500c	7.2	Glycerol-3-phosphate acyltransferase	22	5	1046.5651	1045.5578	1045.5404	0.0175	0	3	2.6	4	K.QSPVSTSALR.L + Deamidation (NQ)
					1435.7166	1434.7093	1434.7554	-0.0460	1	0	5.5	4	R.VAAFWAQAMRLR.D + Oxidation (M)
					1466.7225	1465.7152	1465.7313	-0.0161	1	(1)	5.8	3	K.RFNLSWSIEGTR.S + Deamidation (NQ)
					1659.7863	1658.7790	1658.7973	-0.0183	0	4	2.6	1	K.LGLMSYVADAYLDGR.S + Oxidation (M)
					1909.9203	1908.9130	1908.9792	-0.0662	0	2	3.8	1	R.QVAVDQELIAPAADLAER.R + Deamidation (NQ)

BCG3639	17.1	HTH-type transcriptional repressor KstR	21	2	1648.7782	1647.7709	1647.8072	-0.0363	1	7	0.97	2	R.AMQRNPLLTEAMTR.A + Deamidation (NQ); Oxidation (M)
					1819.8075	1818.8002	1818.7842	0.0160	0	(3)	1.5	1	R.AMANGEPTEDQYHIAR.V + Deamidation (NQ); Oxidation (M)
BCG2891c	11.1	Putative zinc metalloprotease Rv2869c/MT2937	21	3	1127.5760	1126.5687	1126.5481	0.0206	1	1	4.3	1	R.AMYKQATWK.R + Deamidation (NQ)
					1254.6670	1253.6597	1253.7203	-0.0606	1	18	0.07	3	K.VGALVRAIGGGQR.D + Deamidation (NQ)
					1751.8119	1750.8046	1750.8195	-0.0149	0	2	3.9	1	K.VGDTPVSSFDEMAAAVR.K
BCG0602	4.2	GDP-mannose-dependent alpha-mannosyltransferase	20	1	1466.7573	1465.7500	1465.7235	0.0266	0	(19)	0.095	1	R.LQSAMPTAVFTGAR.Y + Deamidation (NQ); Oxidation (M)
BCG3940c	8.8	ESX-2 secretion system protein EccE2	19	3	1287.6403	1286.6330	1286.6691	-0.0360	1	12	0.33	1	R.RLGSDAVAGSAQR.W
					1401.6808	1400.6735	1400.7333	-0.0598	0	4	2.2	1	R.LMIPVTDAGELSR.V
					1795.8774	1794.8701	1794.9376	-0.0675	1	3	4	2	R.RPVKWNDPITLANNR.S + 2 Deamidation (NQ)
BCG0733	5.3	Elongation factor G	19	2	943.6120	942.6047	942.5862	0.0186	1	1	1.9	1	K.IAGSQVLKK.A
					1091.542	1090.535	1090.540	-0.0054	1	14	0.23	1	R.QADKYDVPR.I

					6	3	7						
					1433.734 0	1432.726 7	1432.672 8	0.0539	1	4	2.7	2	R.GQIQAMEERAGAR.V + Deamidation (NQ); Oxidation (M)
BCG3025 c	5.3	Acetolactat e synthase	20	2	1370.726 6		1370.757 2	-0.0306	0	10		1	VALINNGNLGMVR
					2039.025 3		2039.016	0.0093	0	10		1	QNLGMPGMHGTVAAVAALQR + Deamidation (NQ) + Oxidation (M)
BCG1667	4.5	DNA polymerase I	17	2	3388.539 8		3388.623 8	-0.084	0	17		1	AETPQQQQLSLLDDDDTDAETIQTTILRA R + Deamidation (NQ)
					1240.682 3		1240.570 6	0.1117	0			6	QWLAEHAGDGR + Deamidation (NQ)
BCG2921 c	5.1	Hypothetic al protein Mb2924c	17	2	1893.945 8		1893.934 3	0.0115	0	11		1	AVMVSLQRGMQQMGALR + Deamidation (NQ) + Oxidation (M)
					2309.074		2309.123 8	-0.0498	0	6		1	GMIGKPGAGVCPVRGHSNVQGDR + Deamidation (NQ) + Oxidation (M)
BCG1855	3.5	Probable acetolactat e synthase		2	1459.708 7		1459.659 4	0.0493	0	9		1	STDTAPAQTMHAGR + Oxidation (M)
					2028.028 7		2028.011 4	0.0173	0	4		1	MSTDTAPAQTMHAGRLIAR
BCG3770	6.6	2-isopropylm alate synthase	37	7	1637.924 1		1637.774	0.1501	0	2		1	CVEQAAKYPGTQWR + Deamidation (NQ)
					1719.028 1		1718.823 1	0.205	0	10		2	GVDPQIDFSNIDEIR + Deamidation (NQ)
					1719.903 3		1719.807 1	0.0962	0	2		3	GVDPQIDFSNIDEIR + 2Deamidation (NQ)
					2014.107 1		2013.944 7	0.1624	0	1		4	VGQPSWNPQRASSMPVNR + 2Deamidation (NQ)
					907.4709		907.4421	0.0288	0	9		1	YPGTQWR
					944.5059		944.4697	0.0362	0	10		1	NRTWPDR
					1874.929		1874.924	0.0053	0	13		1	GVDPQIDFSNIDEIRR

					6		3						
BCG0391	6.3	Chaperone protein dnaJ1		2	1336.7313		1336.7332	-0.0019	0	2		1	IPPGVEDGQRIR
					1377.7678		1377.625	0.1428	0	12		1	VCPTCNGSGVINR + Carboxymethyl [C]
BCG0992	3.4	Putative DNA-ligase like protein Mb0963		2	1412.6611		1412.7493	-0.0882	0	11		2	LDADAPVADRLTR
					1563.8906		1563.849	0.0416	0	2		1	QLSYDEVLTRIAR
BCG0957	14.8	Probable enoyl-CoA hydratase echA6		3	1799.9275		1799.9497	-0.0222	0	4		6	NALNSQLVEELTQAIR + Deamidation (NQ)
					1956.9415		1957.0349	-0.0934	0	8		1	RNALNSQLVEELTQAIR + Deamidation (NQ)
					2163.0352		2163.0715	-0.0363	0	1		2	ELFDKAWGSQDVIEAQVAR + Deamidation (NQ)
BCG1671	4.7	UvrABC system protein B (UvrB protein)	36	2	1799.8248		1799.8922	-0.0674	0	15		2	QIAYNEANGIDPQPLR + Deamidation (NQ)
					1957.041		1957.1229	-0.0819	0	21		1	QLRLGDYDVLVGINLLR
BCG0976c	7.0	Probable manganese transport protein mntH	33	2	1609.7388		1609.8292	-0.0904	0	16		1	RSPELSHTDGLIER
					1784.8386		1784.9072	-0.0686	0	17		1	SSGIMREHGVQVDTLR
BCG1389c	3.3	Alpha-1,4-glucan:maltose-1-phosphate maltosyltransferase	32	2	1570.8634		1570.9427	-0.0793	0	16		1	SLQPFITRLNIIR
					2024.1165		2024.0083	0.1082	1	17		1	DFASALDQGRSLQPFITR + Deamidation (NQ)
BCG2870c	4.5	Uncharacterized	32	2	1515.7634		1515.7649	-0.0015		13		1	ATVDDVVLGDNELR

		protein Rv2850c/MT2916			1611.77		1611.7584	0.0116		19		2	RMAYEADPDAFVAR
BCG2957	2.4	Phthiocerol synthesis polyketide synthase type I PpsE	30	2	1631.8055		1631.8243	-0.0188		19		1	MSIPENAIAVVGMAGR+Oxidation (M)
					2227.1753		2227.1514	0.0239		11		2	HNVTMAAVVHDREHAATVLR
BCG3443	5.2	Putative diacyglycerol O-acyltransferase Rv3371/MT3481	30	2	989.5697		989.5526	0.0171		13		1	YQAVRVPR + Deamidation (NQ)
					1725.8158		1725.9395	-0.1237		16		1	RVALPRPGDEAELFR

Table S3: Primers used in the study.

Gene		Primers (5'→3')	Melting Temperature	Amplicon length
16S rRNA	FP	TAGGCGTTCCCTTGTGGC	62.18	142
	RP	CAGTCTCTCACGAGTCCC	62.18	
BCG0946	FP	CGCTGTTACGTTCCTGTCC	62.32	212
	RP	GTCCAAGCGTCGGCAGAT	62.18	
BCG2433c	FP	GATGGGCGTCGGTTTCG	62.02	268
	RP	CGTCGCGTTGCTCACAG	62.02	
BCG2654c	FP	GTGCATGGGACGGCTCAA	62.18	248
	RP	ATCTTGACGGTGTGGCGG	62.18	
BCG3756c	FP	GCTGGGTTATCTACGGGTTG	62.45	213
	RP	ATGAGCGCGACGAGGTG	62.02	
BCG3226c	FP	GGTCAGCAGTTTGGTGGAG	62.32	157
	RP	CAGGTCAAACAGCAGTTCAG	60.4	
BCG0932	FP	GGGTGCGTTGGCGATTC	62	104
	RP	CGATGGGGTGTGGGG	61.6	
BCG3932	FP	TAGTGATCGGATCGGTCTTC	60.4	203
	RP	GGGTAGCGGTGAGGGGT	64	
BCG3940c	FP	CGCAGCTAGTCACCGAGAT	62.32	150
	RP	GAATCACGATCCTCTTGGC	60.18	
BCG0332	FP	ATCGGTTGGGAGGCTGGT	62	149
	RP	GTATCGGGCAACTCGGCA	62	

Primers used for the qRT-PCR of the mycobacterial genes.

Primers for cloning VapC48 into pVV16		Primers (5'→3')	Melting Temperature
Forward primer with *NdeI*	FP	ATCTCATATGAGCGAAACCTTTGACG	65
Reverse primer with *HindIII*	RP	ACTCGGATCCAGCCGGAGAAGGGGTC	61
**Primers for cloning IdeR into pVV16**		**Primers (5'→3')**	**Melting Temperature**
Forward primer with *NdeI*	FP	ATATATATGAACGAGCTGGTTGAT	52.2
Reverse primer with *HindIII*	RP	TATAAGCTTTCAGACTTTTTCGACCTT	56.9

Primers used for cloning of VapC48 gene into pVV16 shuttle vector.
